# MORG1 Haploinsufficiency Is Associated with Stage- and Sex-Dependent Renoprotection and Signalling Alterations in Diabetic Kidney Disease

**DOI:** 10.3390/ijms27187990

**Published:** 2026-09-08

**Authors:** Jonas Till Ihle, Luisa Duong, Nadja Ziller, Eric Jankowski, Karin Elflein, Alexander Berndt, Nikolaus Gaßler, Gunter Wolf, Ivonne Loeffler

**Affiliations:** 1 Department of Internal Medicine III, Jena University Hospital, 07747 Jena, Germany; jonas.ihle@gmx.de (J.T.I.); luisa.duong@med.uni-jena.de (L.D.); nadja.ziller@med.uni-jena.de (N.Z.); eric.jankowski@med.uni-jena.de (E.J.); gunter.wolf@med.uni-jena.de (G.W.); 2 Central Experimental Animal Facility, Jena University Hospital, 07747 Jena, Germany; karin.elflein@med.uni-jena.de; 3 Section Pathology, Institute of Forensic Medicine, Jena University Hospital Jena, 07747 Jena, Germany; alexander.berndt@med.uni-jena.de (A.B.); nikolaus.gassler@med.uni-jena.de (N.G.); 4 Thuringian Center for Molecular Diagnostics and Prevention–Center for Translational Medicine (ThIMEDOP-CeTraMed), Jena University Hospital, 07747 Jena, Germany

**Keywords:** diabetic kidney disease, aging, sex differences, disease progression

## Abstract

Type 2 diabetes mellitus (T2DM) is a major age-associated metabolic disease, and aging substantially contributes to the increasing burden and progression of chronic complications, including diabetic kidney disease (DKD). However, the molecular mechanisms underlying changes in renal susceptibility during disease progression remain incompletely understood. We hypothesized that the scaffold protein mitogen-activated protein kinase organizer 1 (MORG1/WDR83) contributes to stage-dependent differences in DKD and that these effects may vary according to biological sex. Heterozygous *Morg1* deletion was introduced into the diabetic C57BLKS/J *db/db* mouse model, and male and female mice were analysed at early and later disease stages alongside non-diabetic controls. Renal fibrosis, albuminuria, HIF-1α expression, mTORC1 and ERK1/2 activity, and renal cortical gene expression were assessed. *Morg1* haploinsufficiency was associated with reduced renal fibrosis and albuminuria during early diabetes, whereas these protective effects were less evident in later-stage diabetic animals. HIF-1α expression decreased during progression in most groups but was relatively preserved in later-stage female *Morg1* heterozygotes, whereas mTORC1 and ERK1/2 signalling was altered in a genotype- and stage-dependent manner. These findings identify MORG1 as a context-dependent modifier of DKD whose renoprotective effects change across disease progression, linking MORG1-associated signalling to the evolving renal phenotype of an age-associated metabolic disease.

## 1. Introduction

Type 2 diabetes mellitus (T2DM) is a major age-associated metabolic disease whose prevalence increases markedly with increasing age. Diabetic kidney disease (DKD) is one of the most common microvascular complications and the leading cause of end-stage renal disease (ESRD) worldwide [1,2]. With population ageing and the increasing prevalence of metabolic risk factors such as obesity, hypertension, and hyperglycemia, the global burden of T2DM and DKD continues to rise [3,4]. Importantly, ageing not only increases exposure to the diabetic milieu but also alters renal susceptibility to chronic metabolic injury, making the interaction between age and diabetes an important determinant of DKD development and progression.

DKD develops through interconnected processes involving hyperglycemia-induced cellular injury, oxidative stress, inflammation, and progressive fibrosis. Chronic hyperglycemia promotes the formation of advanced glycation end products (AGEs), podocyte and endothelial dysfunction, and the activation of profibrotic pathways, including transforming growth factor-β (TGF-β)/Smad and MAPK signalling, ultimately leading to extracellular matrix (ECM) accumulation and glomerulosclerosis [5,6,7].

Age and sex are major biological modifiers of DKD susceptibility and progression [8,9]. Ageing is accompanied by structural and functional alterations of the kidney that may increase its vulnerability to metabolic stress and contribute to ECM accumulation, fibrosis, and the progressive loss of renal function [8,10]. In parallel, sex differences arise from hormonal, chromosomal, and gene-regulatory mechanisms and vary across DKD phenotypes and diabetes types. Male sex is more consistently associated with albuminuria, whereas findings regarding disease progression are less uniform [9,11,12,13,14]. The combined effects of ageing, biological sex, and prolonged metabolic stress may therefore contribute substantially to the heterogeneity of DKD.

Several pathways implicated in DKD, including hypoxia-associated signalling, MAPK/ERK signalling, and nutrient-sensing pathways such as mTORC1, are influenced by age, sex, and disease progression [15]. A molecular scaffold involved in the regulation of these pathways is mitogen-activated protein kinase organizer 1 (MORG1/WDR83), a WD40-repeat protein that facilitates ERK/MAPK signalling by promoting the assembly of signalling complexes [16]. In the kidney, MORG1 interacts with prolyl hydroxylase domain 3 (PHD3), thereby influencing hypoxia-inducible factor (HIF) stability and downstream hypoxia-associated, inflammatory, and fibrotic responses [17,18].

Previous studies have demonstrated that heterozygous MORG1 deficiency attenuates diabetic nephropathy, reduces tubulointerstitial fibrosis, and suppresses epithelial-to-mesenchymal transition-like changes in diabetic mice [19,20]. Reduced MORG1 expression also protects against renal inflammation in experimental endotoxemia through the modulation of HIF-2α and NF-κB signalling [21]. More recently, MORG1 was identified as a negative regulator of mammalian target of rapamycin complex 1 (mTORC1) that limits Rag-dependent lysosomal recruitment under nutrient-rich conditions [22,23]. In addition, the MORG1/WDR83 locus produces the natural antisense transcripts WDR83os and DHPS (deoxyhypusine synthase), the latter of which regulate MORG1 expression through a bidirectional feedback mechanism [24].

Despite the established role of MORG1 in renal injury and fibrosis, little is known about how ageing, biological sex, and disease progression influence MORG1-associated effects in DKD. We hypothesized that partial MORG1 deficiency is associated with sex- and stage-dependent differences in DKD and fibrosis-associated signalling pathways. By examining male and female mice at early and later stages of diabetes, we aimed to determine whether the renoprotective effects of reduced MORG1 expression are maintained across these experimental stages. Our findings demonstrate that MORG1 haploinsufficiency is associated with a protective renal phenotype during early DKD, whereas this protective phenotype is less evident in later-stage diabetic animals.

## 2. Results

### 2.1. Morg1 Haploinsufficiency Does Not Alter Baseline Metabolic Characteristics

Compared with nondiabetic controls, all diabetic animals were obese and hyperglycemic. Body weight and fasting blood glucose increased with disease progression, which was consistent with the *db/db* phenotype. Among non-diabetic animals, males were heavier than females, whereas this difference was not detected under diabetic conditions. Fasting blood glucose levels were comparable between the sexes. Heterozygous *Morg1* deletion did not affect body weight compared with wild-type mice. Fasting blood glucose levels were numerically lower in heterozygous mice with both early- and late-stage diabetes, although these differences did not consistently reach statistical significance. A statistically significant difference was observed only in female heterozygous mice during the early stage of diabetes. Castration did not alter body weight or fasting blood glucose within either sex, although diabetic castrated females presented lower fasting glucose levels than diabetic castrated males did (Table 1).

### 2.2. Renal MORG1-Associated Gene Expression Exhibits Sex- and Stage-Dependent Differences

The transcription levels of *Morg1* and its natural antisense transcripts (NATs), *Wdr83os* and *Dhps*, were quantified in renal cortex homogenates (Table 2) [24].

In wild-type animals, *Morg1* expression remained largely stable across sex, diabetic status, and disease stage. As expected, *Morg1* mRNA levels were markedly reduced in heterozygous mice, indicating a clear genotype effect. *Wdr83os* showed only minor variation across conditions. In contrast, *Dhps* exhibited the greatest variation according to sex and disease stage, with higher basal expression in wild-type females and reduced expression at later diabetic stages. *Dhps* expression was numerically lower in *Morg1* heterozygous animals, particularly in females, although these differences did not reach statistical significance.

Prepubertal gonadectomy affected the expression of all three transcripts. *Morg1* and *Dhps* generally decreased after castration, whereas *Wdr83os* showed sex-dependent changes, resulting in a significant castration × sex interaction via multiway ANOVA. These findings support an association between gonadal hormone status and expression of the *Morg1*/*Wdr83os*/*Dhps* locus.

### 2.3. Morg1 Haploinsufficiency Is Associated with Reduced Early Renal Injury, but This Difference Is Not Detected at Later Disease Stages

**Figure 1:**
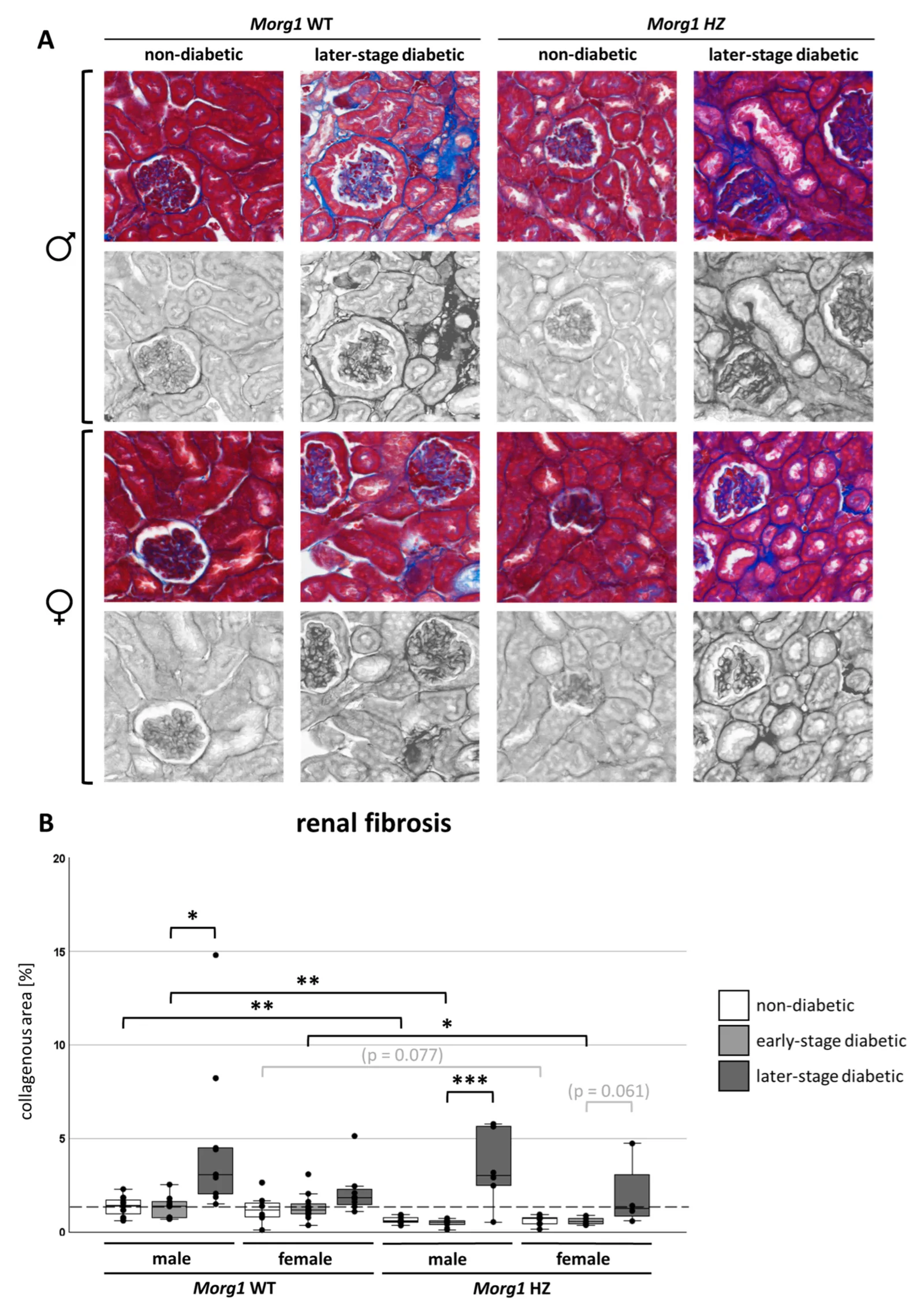
Analysis of renal fibrosis via Azan trichrome staining. (**A**) Representative images of non-diabetic and late-stage diabetic groups. Magnification: 400×. (**B**) Quantification of collagen blue areas is shown as a percentage of the whole cortical area. The dashed line indicates the median value of the non-diabetic *Morg1* WT male group and is shown as a reference. Significance levels: * *p* < 0.05, ** *p* < 0.01, *** *p* < 0.001. Summary of the results of the multiway ANOVA analysis is provided in Supplementary Table S4. Sample sizes (from left to right) were as follows: male, non-diabetic *Morg1* WT, *n* = 11; male, diabetic *Morg1* WT, early-stage, *n* = 10; male, diabetic *Morg1* WT, later-stage, *n* = 9; female, non-diabetic *Morg1* WT, *n* = 8; female, diabetic *Morg1* WT, early-stage, *n* = 12; female, diabetic *Morg1* WT, later-stage, *n* = 8; male, non-diabetic *Morg1* HZ, *n* = 8; male, diabetic *Morg1* HZ, early-stage, *n* = 9; male, diabetic *Morg1* HZ, later-stage, *n* = 6; female, non-diabetic *Morg1* HZ, *n* = 8; female, diabetic *Morg1* HZ, early-stage, *n* = 5; female, diabetic *Morg1* HZ, later-stage, *n* = 4 *Morg1*: mitogen-activated protein kinase organizer 1, WT: wild-type, HZ: heterozygous.

As the primary endpoint of the study, renal fibrosis was quantified in Azan-stained kidney sections via digital image analysis (Figure 1).

Fibrosis did not differ between non-diabetic and early-stage diabetic wild-type diabetic mice. At the late stage, however, collagen deposition increased, most prominently in males, resulting in higher fibrosis levels than those in corresponding female animals. Multiple-way ANOVA confirmed a significant sex × disease stage interaction.

*Morg1* heterozygous mice presented significantly lower collagen deposition under both non-diabetic and early diabetic conditions, confirming previous findings [19]. This genotype effect was no longer evident at the later disease stage, with multiway ANOVA confirming a significant genotype × disease stage interaction.

Castrated animals exhibit diabetes-dependent regulation of fibrosis. Non-diabetic castrated animals presented increased ECM accumulation, whereas early-stage diabetic castrated animals showed reduced ECM deposition. Multiple-way ANOVA confirmed a significant diabetes × castration interaction (Figure S1).

**Figure 2:**
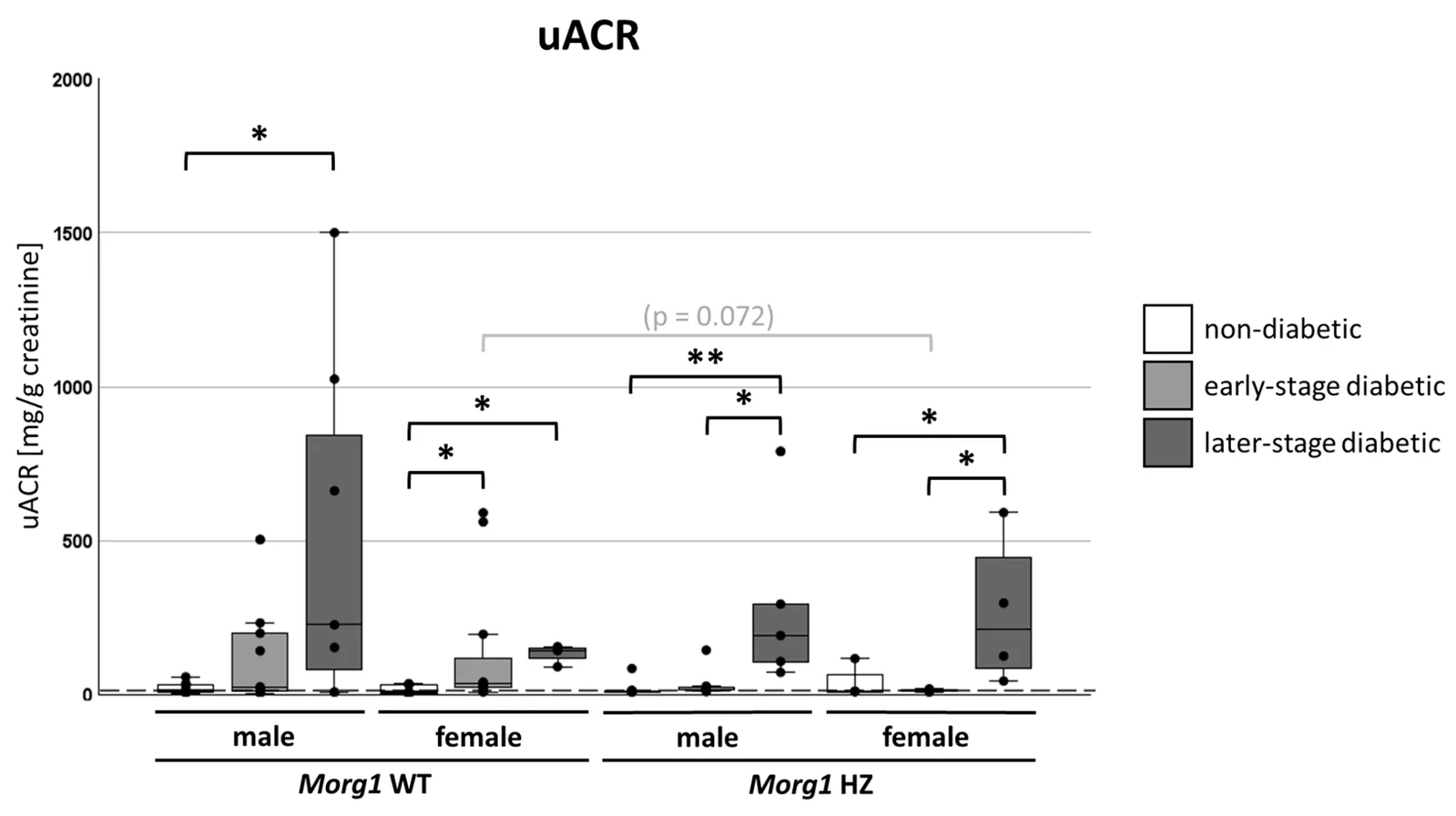
Analysis of the urinary albumin-to-creatinine ratio (uACR). Normoalbuminuria: <30 mg/g creatinine; microalbuminuria: 30–300 mg/g creatinine; macroalbuminuria: >300 mg/g creatinine. The dashed line indicates the median value of the non-diabetic *Morg1* WT male group and is shown as a reference. Significance levels: * *p* < 0.05, ** *p* < 0.01. Summary of the results of the multiway ANOVA analysis is provided in Supplementary Table S4. Sample sizes (from left to right) were as follows: male, non-diabetic *Morg1* WT, *n* = 11; male, diabetic *Morg1* WT, early-stage, *n* = 10; male, diabetic *Morg1* WT, later-stage, *n* = 7; female, non-diabetic *Morg1* WT, *n* = 8; female, diabetic *Morg1* WT, early-stage, *n* = 11; female, diabetic *Morg1* WT, later-stage, *n* = 3; male, non-diabetic *Morg1* HZ, *n* = 8; male, diabetic *Morg1* HZ, early-stage, *n* = 8; male, diabetic *Morg1* HZ, later-stage, *n* = 5; female, non-diabetic *Morg1* HZ, *n* = 4; female, diabetic *Morg1* HZ, early-stage, *n* = 5; female, diabetic *Morg1* HZ, later-stage, *n* = 4. *Morg1*: mitogen-activated protein kinase organizer 1, WT: wild-type, HZ: heterozygous.

The urinary albumin-to-creatinine ratio (uACR) was determined as a measure of renal injury (Figure 2). Early-stage diabetic wild-type mice exhibited increased uACR compared with non-diabetic controls. No clear sex differences were observed at this stage. With prolonged diabetes, the uACR increased further, with numerically higher values in males.

Non-diabetic *Morg1* heterozygous mice showed no marked differences from wild-type controls. In agreement with previous studies [19], early-stage diabetic heterozygotes remained largely normoalbuminuric, indicating attenuation of diabetes-induced albuminuria. At later stages, however, the uACR also increased in heterozygous animals, reaching levels comparable to those of wild-type males. The early-stage difference in albuminuria associated with *Morg1* haploinsufficiency was no longer evident at the later disease stage, with multiway ANOVA confirming a significant genotype × disease-stage interaction.

In castrated males, the uACR was already moderately elevated under non-diabetic conditions and remained largely unchanged with diabetes. In contrast, ovariectomized females showed greater variability, with diabetic animals displaying the highest uACR values (Figure S2).

### 2.4. HIF-1α Signalling Shows Distinct Genotype-, Sex-, and Stage-Associated Patterns

**Figure 3:**
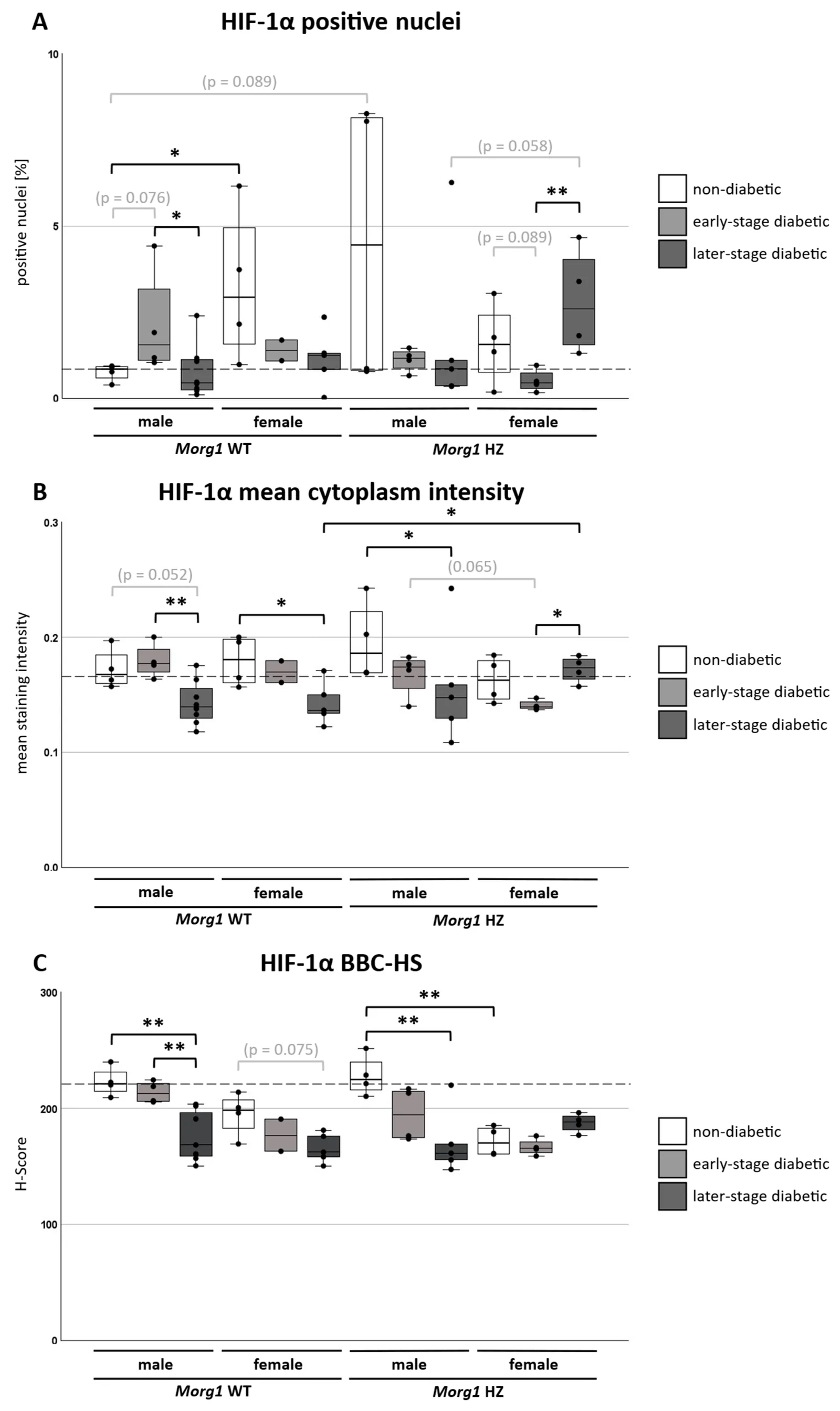
Analysis of HIF-1α protein expression was quantified as the percentage of positive nuclei (**A**), mean cytoplasmic intensity (**B**) and blue–brown color H-score (**C**). Dashed lines indicate the median value of the corresponding non-diabetic *Morg1* WT male group and are shown for reference. Significance levels: * *p* < 0.05, ** *p* < 0.01. Summary of the results of the multiway ANOVA analysis is provided in Supplementary Table S4. Sample sizes (from left to right) were as follows: male, non-diabetic *Morg1* WT, *n* = 4; male, diabetic *Morg1* WT, early-stage, *n* = 4; male, diabetic *Morg1* WT, later-stage, *n* = 8; female, non-diabetic *Morg1* WT, *n* = 4; female, diabetic *Morg1* WT, early-stage, *n* = 2; female, diabetic *Morg1* WT, later-stage, *n* = 5; male, non-diabetic *Morg1* HZ, *n* = 4; male, diabetic *Morg1* HZ, early-stage, *n* = 4; male, diabetic *Morg1* HZ, later-stage, *n* = 5; female, non-diabetic *Morg1* HZ, *n* = 4; female, diabetic *Morg1* HZ, early-stage, *n* = 4; female, diabetic *Morg1* HZ, later-stage, *n* = 4. HIF-1α: hypoxia-inducible factor 1-alpha, *Morg1*: mitogen-activated protein kinase organizer 1, BBC-HS: blue–brown color H-score, WT: wild-type, HZ: heterozygous.

Renal HIF-1α expression was assessed by the percentage of positive nuclei, mean cytoplasmic intensity, and blue–brown color H-score (BBC-HS; Figure 3A–C) [25].

In wild-type males, nuclear HIF-1α positivity increased during early diabetes but decreased at later stages. This decline was also reflected by reduced cytoplasmic intensity and BBC-HS, indicating an overall reduction in HIF-1α expression with prolonged diabetes. Wild-type females exhibited higher basal nuclear HIF-1α positivity than males, but all three readouts progressively declined during disease progression.

In heterozygous animals, the basal sex pattern was reversed. Male and female heterozygous mice showed different numerical patterns across disease stages, with comparatively higher HIF-1α levels observed in late-stage female heterozygous animals across all three readouts. Overall, advanced diabetic disease was associated with reduced HIF-1α expression in most groups, whereas relative preservation was observed in female *Morg1* heterozygotes.

PHD3 expression was quantified by the mean cytoplasmic staining intensity (Figure S3). Consistent with previous findings [17,19], non-diabetic *Morg1* heterozygous mice presented reduced PHD3 expression. In diabetic heterozygous animals, numerically higher PHD3 levels were observed in females than in males at both disease stages, reaching values comparable to those of wild-type females.

### 2.5. MORG1 Deficiency Is Associated with Reciprocal Alterations in mTORC1 and ERK Signalling

**Figure 4:**
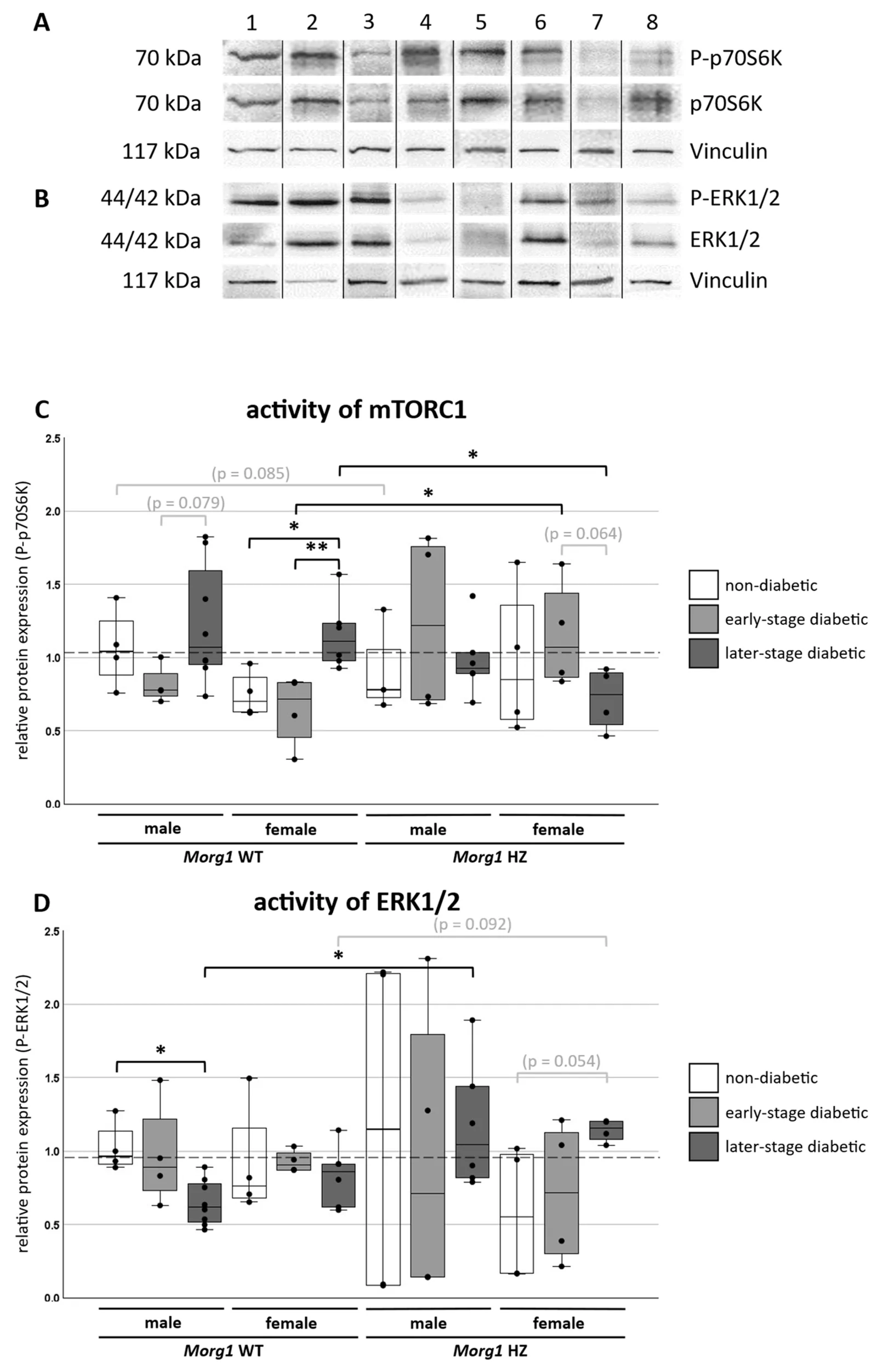
Analysis of mTORC1 (**A**,**C**) and ERK1/2 (**B**,**D**) in the renal cortex. Representative Western blots (**A**,**B**) were selected from the biological replicates included in the quantitative analyses (**C**,**D**). Lane 1: male non-diabetic *Morg1* WT; Lane 2: male late-stage *Morg1* WT; Lane 3: female non-diabetic *Morg1* WT; Lane 4: female late-stage *Morg1* WT; Lane 5: male non-diabetic *Morg1* HZ; Lane 6: male late-stage *Morg1* HZ; Lane 7: female non-diabetic *Morg1* HZ; Lane 8: female late-stage *Morg1* HZ. Protein phosphorylation levels (P-p70S6K for mTORC1; P-ERK1/2 for ERK activity) are shown as relative densitometric values normalized to the mean of the corresponding non-diabetic wild-type male group. Dashed lines indicate the median value of the corresponding non-diabetic *Morg1* WT male group and are shown for reference. Significance levels: * *p* < 0.05, ** *p* < 0.01. Summary of the results of the multiway ANOVA analysis is provided in Supplementary Table S4. Sample sizes (from left to right) were as follows: male, non-diabetic *Morg1* WT, *n* = 4; male, diabetic *Morg1* WT, early-stage, *n* = 4; male, diabetic *Morg1* WT, later-stage, *n* = 8; female, non-diabetic *Morg1* WT, *n* = 4; female, diabetic *Morg1* WT, early-stage, *n* = 4; female, diabetic *Morg1* WT, later-stage, *n* = 6; male, non-diabetic *Morg1* HZ, *n* = 3; male, diabetic *Morg1* HZ, early-stage, *n* = 4; male, diabetic *Morg1* HZ, later-stage, *n* = 6; female, non-diabetic *Morg1* HZ, *n* = 4; female, diabetic *Morg1* HZ, early-stage, *n* = 4; female, diabetic *Morg1* HZ, later-stage, *n* = 4. ERK1: extracellular signal-regulated kinase 1, mTORC1: mechanistic target of rapamycin complex 1, *Morg1*: mitogen-activated protein kinase organizer 1, WT: wild-type, HZ: heterozygous.

mTORC1 activity was assessed by the ratio of phosphorylated to total p70S6K protein (Figure 4C). Male animals showed only minor diabetes-associated changes in mTORC1 activity irrespective of genotype. In contrast, female wild-type mice exhibited significantly increased activity at later disease stages, whereas this increase was absent in heterozygous animals. Overall, mTORC1 activity showed different stage-dependent patterns according to genotype, with multiway ANOVA confirming a significant diabetes duration × genotype interaction. This pattern was most apparent in female animals.

ERK/MAPK signalling was evaluated by the ratio of phosphorylated to total ERK1/2 protein (Figure 4D). In wild-type males, phosphoERK1/2 levels were lower in late-stage diabetic animals than in non-diabetic controls, whereas this decline was not observed in *Morg1* heterozygous mice, resulting in significantly greater ERK activity at late stages than in wild-type animals. A numerically similar pattern was observed in females, although the corresponding difference did not reach statistical significance (*p* = 0.092). Despite considerable interindividual variability, multiway ANOVA revealed a significant main effect of *Morg1* genotype.

### 2.6. MORG1-Associated Signals Localize to Multiple Renal Compartments During DKD

**Figure 5:**
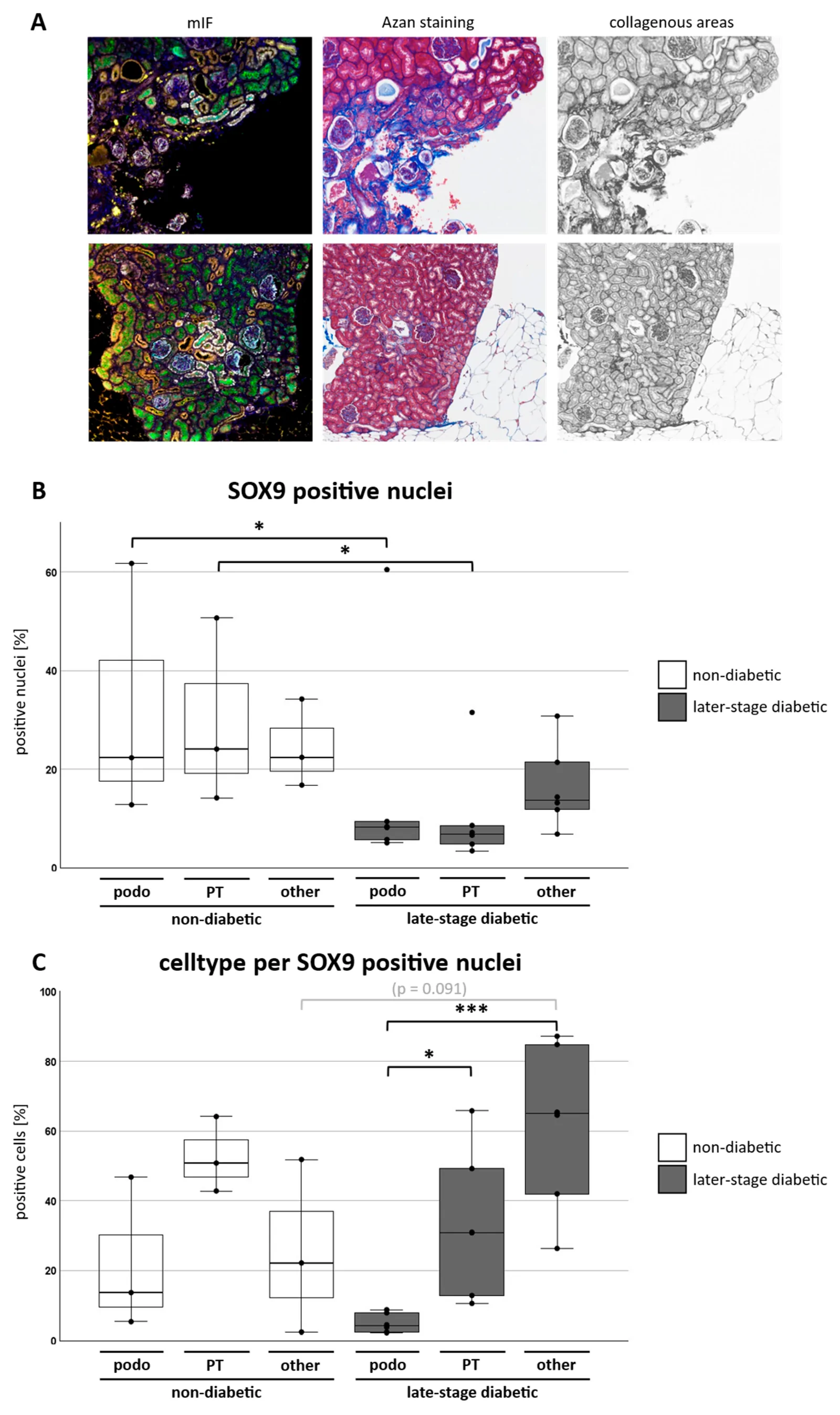
Multiplex immunofluorescence (mIF) analysis of MORG1-associated signals in late-stage diabetic wild-type male kidneys. Multiplex immunofluorescence analyses were exploratory. (**A**) Representative mIF staining of Synaptopodin (magenta), GGT1 (green), MORG1 (blue), DHPS (yellow) and WDR83os (orange) compared with Azan staining and collagenous areas in two different kidney samples. (**B**) Percentage of SOX9-positive cells within each cell class. (**C**) Percentage contribution of each cell class to the total SOX9-positive population. Significance levels: * *p* < 0.05, *** *p* < 0.001. Summary of the results of the multiway ANOVA analysis is provided in Supplementary Table S4. Sample sizes were = 3, 3, 3, 6, 6, and 6 for the groups shown from left to right, respectively. SOX9: SRY-box transcription factor 9, podo: podocytes, PT: proximal tubule cells.

Multiplex immunofluorescence was performed to assess the cellular localization of MORG1, WDR83os, DHPS, and SOX9 (Figure 5).

MORG1-, WDR83os-, and DHPS-associated signals were detected in multiple renal cell populations. SOX9-positive cells were observed in both non-diabetic and diabetic kidneys but appeared redistributed toward non-podocyte/non-proximal tubular compartments in late-stage disease (Figure 5A,B). In areas of advanced diabetic injury, SOX9- and MORG1-associated signals frequently colocalized with regions of interstitial remodelling, suggesting a potential association with fibrosis-related cellular states (Figure 5C).

Multiplex immunofluorescence was performed as an exploratory analysis. The spatial distribution and colocalization patterns are presented qualitatively (Figure 5A), whereas the predefined cell-type proportions shown in Figure 5B,C were quantified. Given the small group sizes, these quantitative analyses should be considered exploratory and hypothesis-generating.

## 3. Discussion

We hypothesized that MORG1 acts as a context-dependent modifier of diabetic kidney disease, with its effects varying according to biological sex and experimental disease stage. Our findings support this hypothesis. *Morg1* haploinsufficiency was associated with reduced renal fibrosis and albuminuria during early-stage diabetes, whereas this protective phenotype was no longer evident at the later disease stage. This stage-dependent difference was supported by significant genotype × disease stage interactions for both renal fibrosis and albuminuria [19]. In addition, *Morg1* haploinsufficiency was associated with distinct sex- and stage-associated patterns in HIF-1α, mTORC1 and ERK signalling. Together, these findings identify *Morg1* haploinsufficiency as being associated with context-dependent differences in DKD phenotypes and signalling across disease stages.

The phenotypic data revealed that reduced *Morg1* expression is associated with less severe diabetic kidney injury during early-stage diabetes, whereas this difference is no longer evident at later disease stages. In wild-type diabetic mice, fibrosis and albuminuria increase with disease progression, with particularly pronounced changes in males. This observation is consistent with previous reports demonstrating a more severe diabetic phenotype in male rodents [13,26]. In contrast, *Morg1* heterozygous mice exhibited reduced collagen deposition under non-diabetic and early diabetic conditions and were largely protected from the early diabetes-induced increase in the uACR. These findings are in line with previous studies showing that *Morg1* haploinsufficiency stabilizes HIF signalling, reduces inflammation, attenuates NF-κB activation, and suppresses epithelial-to-mesenchymal transition-like changes in the diabetic kidney [19,21]. However, this benefit was less evident in later-stage diabetic animals. Because chronological age and diabetes duration are intrinsically linked in our experimental design, this observation cannot be attributed specifically to ageing, prolonged metabolic exposure, DKD progression, or any one of these factors independently [18,19,27].

A second important observation was the presence of sex-associated differences in several renal phenotypes. Male wild-type mice developed more pronounced fibrosis than female mice did, with a significant sex × disease stage interaction supporting differential stage-dependent fibrosis between the sexes. In contrast, sex-related patterns observed for other endpoints were not consistently supported by formal interaction testing and should therefore be interpreted descriptively. The results of the castration experiments further support the contribution of gonadal hormones to MORG1-associated renal phenotypes, which is consistent with previous studies demonstrating hormone-dependent modulation of diabetic kidney injury [28,29]. Because estrogen receptors directly regulate PHD2 and PHD3 transcription [30,31], interactions between sex hormones and the PHD/HIF axis may be particularly relevant in the context of *Morg1* haploinsufficiency. The translation of these sex-associated findings to human DKD requires caution. Sex differences in human DKD reflect a complex interplay of biological, hormonal, behavioural, and metabolic factors that cannot be fully reproduced in experimental models. In particular, *db/db* mice exhibit marked reproductive and endocrine abnormalities, including hypogonadism, which may influence sex-associated renal phenotypes [32]. Moreover, behavioural and lifestyle-related determinants of DKD in humans are inherently absent from this model [33,34]. Thus, the present findings provide insight into sex-associated differences within the *db/db* model but should not be interpreted as directly reproducing the determinants of sex differences observed in human DKD.

A particularly striking finding was the relative preservation of HIF-1α signalling in late-stage female *Morg1* heterozygous mice. This observation is notable because HIF-1α expression generally decreases with disease progression across most groups. In wild-type animals, females presented greater basal nuclear HIF-1α positivity than males did, whereas this sex-associated pattern was reversed in heterozygous animals. Female *Morg1* heterozygotes subsequently showed relative preservation of HIF-1α signalling at later disease stages across all three HIF readouts. One possible hypothesis is that this sex-associated pattern reflects interactions between *Morg1* haploinsufficiency and estrogen-dependent regulation of the PHD/HIF axis. Both ERα and ERβ have been linked to the transcriptional regulation of PHD family members, with ERα increasing PHD3 expression and ERβ regulating PHD2 [30,31]. Therefore, female animals may experience a greater reduction in effective PHD-mediated HIF regulation when *Morg1* expression is reduced. In contrast, male *db/db* mice generally develop a more severe metabolic and inflammatory phenotype [35], which may impair HIF-1α stabilization under diabetic conditions [36]. Although these mechanisms remain speculative, they provide a biologically plausible framework for the observed sex-associated patterns in HIF signalling.

In diabetic wild-type mice, mTORC1 activity increased with disease progression, which is consistent with previous reports describing mTORC1 hyperactivation in diabetic kidneys [37,38,39]. Interestingly, mTORC1 activity was reduced rather than increased in diabetic *Morg1* heterozygous animals, despite the recent description of MORG1 as a negative regulator of Rag-dependent mTORC1 activation [22,23]. One possible hypothesis is that indirect effects associated with *Morg1* haploinsufficiency, including alterations in HIF signalling, inflammation, and tissue homeostasis, may modify the relationship between MORG1 and Rag GTPase-mediated mTORC1 regulation [19,21]. In addition, a partial reduction in *Morg1* expression may preferentially affect PHD3/HIF signalling while leaving other MORG1-dependent functions only partially altered. The observation that reduced mTORC1 activity was most evident in female heterozygous animals is consistent with, but does not establish, a potential interaction between sex-dependent HIF signalling and nutrient-sensing pathways.

Conversely, ERK activity decreased with disease progression in wild-type animals but remained preserved or increased in *Morg1* heterozygous mice. This observation appears counterintuitive because MORG1 was originally identified as a scaffold facilitating ERK pathway activation [16]. However, the reciprocal behavior of ERK and mTORC1 is compatible with the well-described feedback relationship between these pathways. Reduced mTORC1 activity may relieve S6K1-dependent inhibition of upstream signalling, thereby promoting compensatory ERK activation [40,41]. Furthermore, ERK and mTOR signalling are interconnected at several levels, including the regulation of TSC2 and other upstream signalling components [42,43,44]. Additional compensation by alternative scaffold proteins may further contribute to maintaining ERK signalling despite reduced *Morg1* expression [45,46]. Together, these findings indicate that coordinated alterations in mTORC1 and ERK signalling are associated with *Morg1* haploinsufficiency, although the underlying mechanistic relationships remain to be established.

Interestingly, pronounced phenotypic and signalling differences occurred despite only modest changes in *Morg1* transcript abundance. This observation is consistent with the established role of MORG1 as a scaffold protein and suggests that its biological relevance in DKD may not be reflected solely by changes in transcript abundance [16,17,47]. At the transcript level, *Dhps* displayed the most pronounced variation according to sex and stage among the analysed genes, whereas *Wdr83os* remained relatively stable. Given the bidirectional regulatory relationship between MORG1 and DHPS previously described in gastric cancer [24], these findings raise the possibility of an additional regulatory layer within the MORG1/WDR83 locus, whose functional relevance in DKD remains to be established. Although the functional relevance of these NATs in diabetic kidneys remains unclear, their sex-associated expression patterns warrant further investigation.

Multiplex immunofluorescence analyses have extended these findings to the cellular level. MORG1-, WDR83os-, and DHPS-associated signals were detected across multiple renal cell populations, indicating that MORG1-associated biology is not restricted to a single renal compartment. In late-stage diabetic kidneys, SOX9-positive cells were enriched in non-podocyte/non-proximal tubular compartments and frequently colocalized with MORG1-associated signals in areas of interstitial remodelling. Given the established role of SOX9 in injury-responsive tubular cell states and epithelial repair processes [48,49], these observations suggest a spatial association between MORG1-associated signals, injury-responsive cellular states, and areas of fibrotic remodelling during DKD progression. Although the present analyses remain qualitative and the quantitative analyses are exploratory due to the small group sizes, the observed spatial association between SOX9- and MORG1-associated signals provides a rationale for future quantitative investigations of cell type-specific MORG1 signalling during chronic kidney injury.

This study has several strengths, including the combined analysis of genotype, sex, and disease stage, as well as the integration of structural, functional, transcriptional, and signalling-level readouts within one experimental framework. This design allowed us to identify context-dependent effects that would likely have remained undetected in a simpler experimental setting. Several limitations should nevertheless be acknowledged, including reliance on global heterozygous deficiency, as homozygous knockout is embryonically lethal, rather than being based on cell type-specific targeting approaches [50]. Importantly, chronological age and diabetes duration were intrinsically linked in our experimental design, preventing their individual contributions to the observed stage-dependent effects from being distinguished. Thus, our findings demonstrate differences in MORG1-associated renoprotection between early- and later-stage diabetic animals but do not establish whether these differences are attributable to chronological ageing, diabetes duration, DKD progression, or their combined effects. The slightly lower blood glucose levels in heterozygous mice may have contributed to the reduced renal injury observed in female mice during early-stage diabetes; however, this explanation appears unlikely, as a similar numerical difference in glycemia was present during late-stage diabetes without accompanying renoprotection. The gonadectomy experiments were exploratory and cannot fully reproduce the complex endocrine, chromosomal, and developmental determinants of biological sex. Furthermore, the large number of secondary endpoints and prespecified pairwise comparisons without adjustment for multiple testing increase the risk of type I error. Findings from these exploratory analyses should therefore be considered hypothesis-generating and require independent validation. In addition, group sizes varied across secondary analyses because not all biological samples were available from every animal, and partially overlapping cohorts were used. Consequently, subgroup analyses involving genotype, sex, and disease stage may have limited statistical power and should be considered exploratory. Accordingly, the absence of statistical significance in these analyses should not be interpreted as evidence for the absence of a biological effect. Nevertheless, the present findings support a role of MORG1 in modifying diabetic kidney injury and indicate sex- and stage-associated differences in fibrosis- and stress-associated signalling pathways.

In conclusion, *Morg1* haploinsufficiency was associated with reduced renal fibrosis and albuminuria during early-stage diabetes, whereas this protective renal phenotype was less evident at the later disease stage. MORG1-associated signalling patterns differ according to sex and disease stage: HIF-1α expression shows distinct sex- and stage-associated patterns, whereas mTORC1 activity differs across disease stages according to genotype, with this pattern being most apparent in female mice. ERK1/2 activity was associated with the *Morg1* genotype, with descriptively more pronounced differences in male mice. Together, these findings demonstrate that the renal phenotype associated with *Morg1* haploinsufficiency varies between the early and later stages of diabetes and is accompanied by context-dependent alterations in renal stress-associated signalling. Further studies are needed to disentangle the contributions of chronological ageing, diabetes duration, and DKD progression and to determine the underlying cell type-specific mechanisms involved.

## 4. Materials and Methods

### 4.1. Animals

The animals were housed under specific pathogen-free conditions with a 12 h light/dark cycle and free access to food and water. Randomization was not applicable because the animals were allocated according to predefined genotype, sex, diabetic status, and disease stage. The sample size was determined via a priori power analysis. Additional details regarding housing, allocation, and sample availability are provided in the Supplementary Methods and Supplementary Table S5.

### 4.2. RNA Extraction, cDNA Synthesis, and Real-Time PCR

RNA isolation, cDNA synthesis, and semiquantitative RT–PCR were performed as described previously [51]. The primer sequences and annealing temperatures are listed in Table S1. Relative expression levels were calculated via the ΔΔCT method, with *Hprt1* and *Gapdh* used as reference genes. The expression levels were normalized to those of non-diabetic wild-type young males.

### 4.3. Azan Staining

Paraffin-embedded kidney sections (3 µm) were stained using the HEIDENHAIN AZAN Kit (Morphisto, Offenbach am Main, Germany). Whole-slide images were analysed in QuPath version 0.5.1. The detailed image acquisition and analysis settings are provided in the Supplementary Methods.

### 4.4. Immunohistochemistry

Paraffin sections (3 µm) were subjected to heat-mediated antigen retrieval and were stained with antibodies against HIF-1α or PHD3 (Table S2). Detection was performed using DAB and hematoxylin counterstaining.

The quantification of HIF-1α and PHD3 was performed in QuPath version 0.7.0 via custom scripts. The detailed image analysis parameters are described in the Supplementary Methods.

### 4.5. Multiplex Immunofluorescence

Multiplex immunofluorescence was performed as described in the Supplementary Methods. For detailed staining parameters and antibodies see Supplementary Table S3. Image analysis was performed in QuPath version 0.7.0 via a custom classifier.

### 4.6. Western Blot

Protein lysates were prepared via the Complete Lysis M Kit (Roche, Basel, Switzerland) supplemented with a phosphatase inhibitor cocktail (Cell Signaling Technology, Danvers MA, USA). Western blotting was performed as described previously [52]. The primary antibodies used are listed in Table S2. For ERK1/2 and p70S6K analyses, phosphorylated and total protein as well as vinculin were detected sequentially on the same membrane, with stripping between antibody incubations. For densitometric analysis, the phosphorylated-to-total protein ratio was first calculated for each sample (p-ERK1/2/total ERK1/2 or p-p70S6K/total p70S6K). The resulting ratio was subsequently normalized to the corresponding vinculin signal. To account for inter-blot variability and enable comparison across independent Western blot runs, the resulting values were finally normalized to the value obtained from the same internal reference sample, consisting of lysate from a defined mouse that was included on each membrane. The internal reference sample was analyzed and normalized using the same calculation procedure. Thus, the final value was calculated as [(phosphorylated protein/total protein)/vinculin] relative to the corresponding internal reference sample.

### 4.7. Urine Analysis

Urinary creatinine (Cayman Chemicals, Ann Arbor, MI, USA) and albumin (CellTrend GmbH, Luckenwalde, Germany) were quantified via ELISA according to the manufacturer’s instructions.

### 4.8. Statistical Analysis

Statistical analyses were performed via IBM SPSS Statistics version 30.0.0.0 (IBM, Armonk, NY, USA). Renal fibrosis was defined as the primary endpoint and formed the basis of the a priori sample size calculation. All other functional, molecular, and signalling readouts were considered secondary exploratory outcomes. Multiway ANOVA was used to assess the main effects and interactions of sex, diabetes, genotype, and disease stage. Variables with a skewed distribution (renal fibrosis, urinary albumin-to-creatinine ratio, and ERK1/2 activity) were log10-transformed before analysis. Pairwise comparisons were performed via Mann–Whitney U tests and included comparisons of genotypes within corresponding sex and disease-stage groups, disease stages within corresponding sex and genotype groups, and sex within corresponding genotype and disease-stage groups. Comparisons of gonadectomized and intact animals were performed within the corresponding experimental groups. Given the exploratory nature of the secondary analyses, no adjustment for multiple testing was applied, and these findings were interpreted as hypothesis-generating. Statistical significance was defined as *p* ≤ 0.05. Exact *p* values are reported where informative; findings with *p* > 0.05 were considered nonsignificant and were not interpreted as statistical trends. Further details on the outcome measures, sample size determination, model diagnostics, and data presentation are provided in the Supplementary Methods.

## Figures and Tables

**Table 1:** Baseline characteristics of animals. Overview of diabetes duration, body weight and fasting blood glucose in all groups.

Sex	Group	T2DM Duration	Weight	Fasting Blood Glucose
male	non-diabetic *Morg1* WT	-	25.6 ± 0.5	5.5 ± 0.5
	diabetic *Morg1* WT	early-stage later-stage	44.3 ± 2.6 55.3 ± 4.2	12.8 ± 2.3 12.9 ± 1.8
female	non-diabetic *Morg1* WT	-	20.1 ± 0.6	6.3 ± 1.3
	diabetic *Morg1* WT	early-stage later-stage	46.1 ± 2.4 54.8 ± 2.8	11.6 ± 2.0 11.7 ± 2.5
male	non-diabetic *Morg1* HZ	-	25.8 ± 1.1	7.4 ± 0.6
	diabetic *Morg1* HZ	early-stage later-stage	41.8 ± 1.7 59.4 ± 5.2	9.7 ± 2.1 9.4 ± 2.5
female	non-diabetic *Morg1* HZ	-	18.2 ± 0.4	6.2 ± 0.5
	diabetic *Morg1* HZ	early-stage later-stage	45.2 ± 1.7 50.6 ± 8.2	5.9 ± 0.5 9.7 ± 2.7
male (orchiectomized)	non-diabetic *Morg1* WT	-	22.2 ± 0.4	5.9 ± 1.5
	diabetic *Morg1* WT	early-stage	37.4 ± 2.0	14.9 ± 1.7
female (ovariectomized)	non-diabetic *Morg1* WT	-	20.2 ± 1.2	5.6 ± 1.1
	diabetic *Morg1* WT	early-stage	43.1 ± 4.3	9.3 ± 2.8

T2DM: type 2 diabetes mellitus, WT: wild-type, HZ: heterozygous. Exact *p* values and the corresponding pairwise comparisons are provided in Supplementary Table S6. Sample sizes (from top to bottom) were as follows: male, non-diabetic *Morg1* WT, *n* = 11; male, diabetic *Morg1* WT, early-stage, *n* = 10; male, diabetic *Morg1* WT, later-stage, *n* = 9; female, non-diabetic *Morg1* WT, *n* = 8; female, diabetic *Morg1* WT, early-stage, *n* = 12; female, diabetic *Morg1* WT, later-stage, *n* = 8; male, non-diabetic *Morg1* HZ, *n* = 8; male, diabetic *Morg1* HZ, early-stage, *n* = 9; male, diabetic *Morg1* HZ, later-stage, *n* = 6; female, non-diabetic *Morg1* HZ, *n* = 8; female, diabetic *Morg1* HZ, early-stage, *n* = 5; female, diabetic *Morg1* HZ, later-stage, *n* = 5; orchiectomized male, non-diabetic *Morg1* WT, *n* = 8; orchiectomized male, diabetic *Morg1* WT, early-stage, *n* = 4; ovariectomized female, non-diabetic *Morg1* WT, *n* = 8; ovariectomized female, diabetic *Morg1* WT, early-stage, *n* = 4.

**Table 2:** mRNA expression of *Morg1*, *Wdr83os*, and *Dhps*. The data are displayed as the means ± standard deviations.

Sex	Group	T2DM Duration	*Morg1*	*Wdr83os*	*Dhps*
male	non-diabetic *Morg1* WT	-	1.04 ± 0.10	1.02 ± 0.06	1.03 ± 0.07
	diabetic *Morg1* WT	early-stage later-stage	1.16 ± 0.12 1.32 ± 0.14	1.20 ± 0.08 1.10 ± 0.09	1.52 ± 0.14 1.23 ± 0.09
female	non-diabetic *Morg1* WT	-	1.20 ± 0.09	1.24 ± 0.11	1.22 ± 0.06
	diabetic *Morg1* WT	early-stage later-stage	1.35 ± 0.20 1.10 ± 0.15	1.48 ± 0.17 1.22 ± 0.10	1.36 ± 0.14 1.02 ± 0.08
male	non-diabetic *Morg1* HZ	-	1.16 ± 0.49	1.75 ± 0.46	1.43 ± 0.35
	diabetic *Morg1* HZ	early-stage later-stage	0.73 ± 0.19 0.56 ± 0.05	1.31 ± 0.25 0.95 ± 0.16	1.34 ± 0.23 1.08 ± 0.10
female	non-diabetic *Morg1* HZ	-	0.50 ± 0.04	1.47 ± 0.25	1.17 ± 0.17
	diabetic *Morg1* HZ	early-stage later-stage	0.38 ± 0.02 0.47 ± 0.03	1.12 ± 0.12 0.87 ± 0.21	0.93 ± 0.07 0.91 ± 0.09
male (orchiectomized)	non-diabetic *Morg1* WT	-	1.25 ± 0.10	1.36 ± 0.14	1.23 ± 0.04
	diabetic *Morg1* WT	early-stage	0.94 ± 0.13	0.97 ± 0.05	0.83 ± 0.10
female (ovariectomized)	non-diabetic *Morg1* WT	-	1.16 ± 0.07	1.28 ± 0.06	1.10 ± 0.07
	diabetic *Morg1* WT	early-stage	0.83 ± 0.04	0.90 ± 0.08	1.13 ± 0.20

T2DM: type 2 diabetes mellitus, WT: wild-type, HZ: heterozygous. Exact *p* values and the corresponding pairwise comparisons are provided in Supplementary Table S6. Sample sizes (from top to bottom) were as follows: male, non-diabetic *Morg1* WT, *n* = 11; male, diabetic *Morg1* WT, early-stage, *n* = 10; male, diabetic *Morg1* WT, later-stage, *n* = 9; female, non-diabetic *Morg1* WT, *n* = 8; female, diabetic *Morg1* WT, early-stage, *n* = 12; female, diabetic *Morg1* WT, later-stage, *n* = 8; male, non-diabetic *Morg1* HZ, *n* = 8; male, diabetic *Morg1* HZ, early-stage, *n* = 9; male, diabetic *Morg1* HZ, later-stage, *n* = 6; female, non-diabetic *Morg1* HZ, *n* = 8; female, diabetic *Morg1* HZ, early-stage, *n* = 5; female, diabetic *Morg1* HZ, later-stage, *n* = 4; orchiectomized male, non-diabetic *Morg1* WT, *n* = 8; orchiectomized male, diabetic *Morg1* WT, early-stage, *n* = 4; ovariectomized female, non-diabetic *Morg1* WT, *n* = 8; ovariectomized female, diabetic *Morg1* WT, early-stage, *n* = 4.

## Data Availability

The datasets generated and analysed during the current study are available from the corresponding author upon reasonable request.

## Supplementary Materials

The following supporting information can be downloaded at https://www.mdpi.com/article/10.3390/ijms27187990/s1.

## Abbreviations

The following abbreviations are used in this manuscript:

AGEs	advanced glycation end products
BBC-HS	blue–brown color H-score
DHPS	deoxyhypusine synthase
DKD	diabetic kidney disease
ECM	extracellular matrix
ESRD	end-stage renal disease
HIF	hypoxia-inducible factor
HZ	heterozygous
MORG1	mitogen-activated protein kinase organizer 1
mTORC1	mammalian target of rapamycin complex 1
NAT	natural antisense transcript
PHD3	prolyl hydroxylase domain 3
Podo	podocytes
PT	proximal tubule cells
SOX9	SRY-box transcription factor 9
T2DM	type 2 diabetes mellitus
TGF-β	transforming growth factor-β
uACR	urinary albumin-to-creatinine ratio
WDR83	WD repeat domain 83; synonym for MORG1
WDR83os	natural antisense transcript of WDR83
WT	wild-type
