# Supplementary material for: MORG1 Haploinsufficiency Is Associated with Stage- and Sex-Dependent Renoprotection and Signalling Alterations in Diabetic Kidney Disease
Source: Int J Mol Sci. 2026 Sep 8;27(18):7990. doi: 10.3390/ijms27187990 (PMC13606655; doi:10.3390/ijms27187990)
Supplement: Supplementary file 1 [file ijms-27-07990-s001.zip › Supplementary Figures and Tables.pdf]

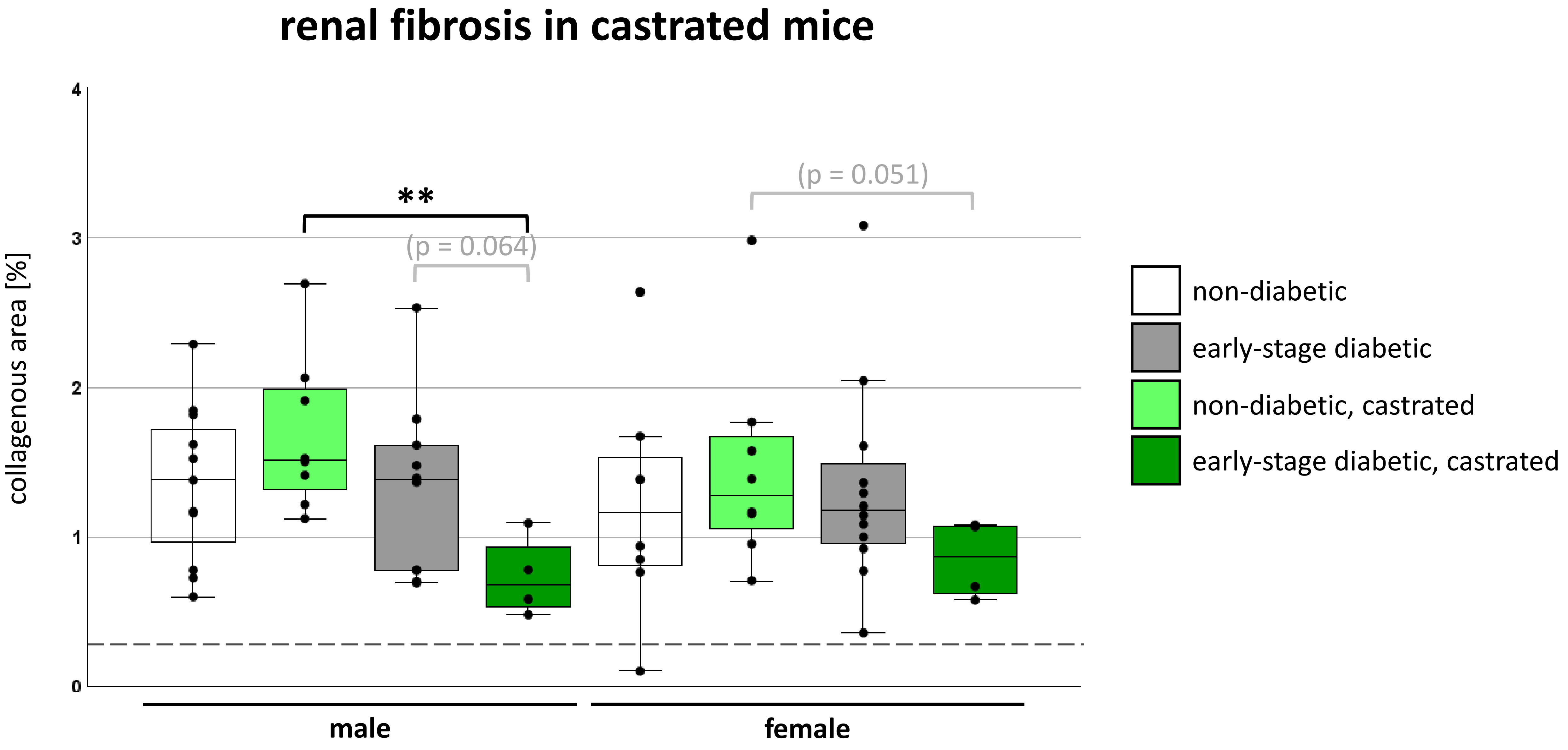

Supplementary Figure S1

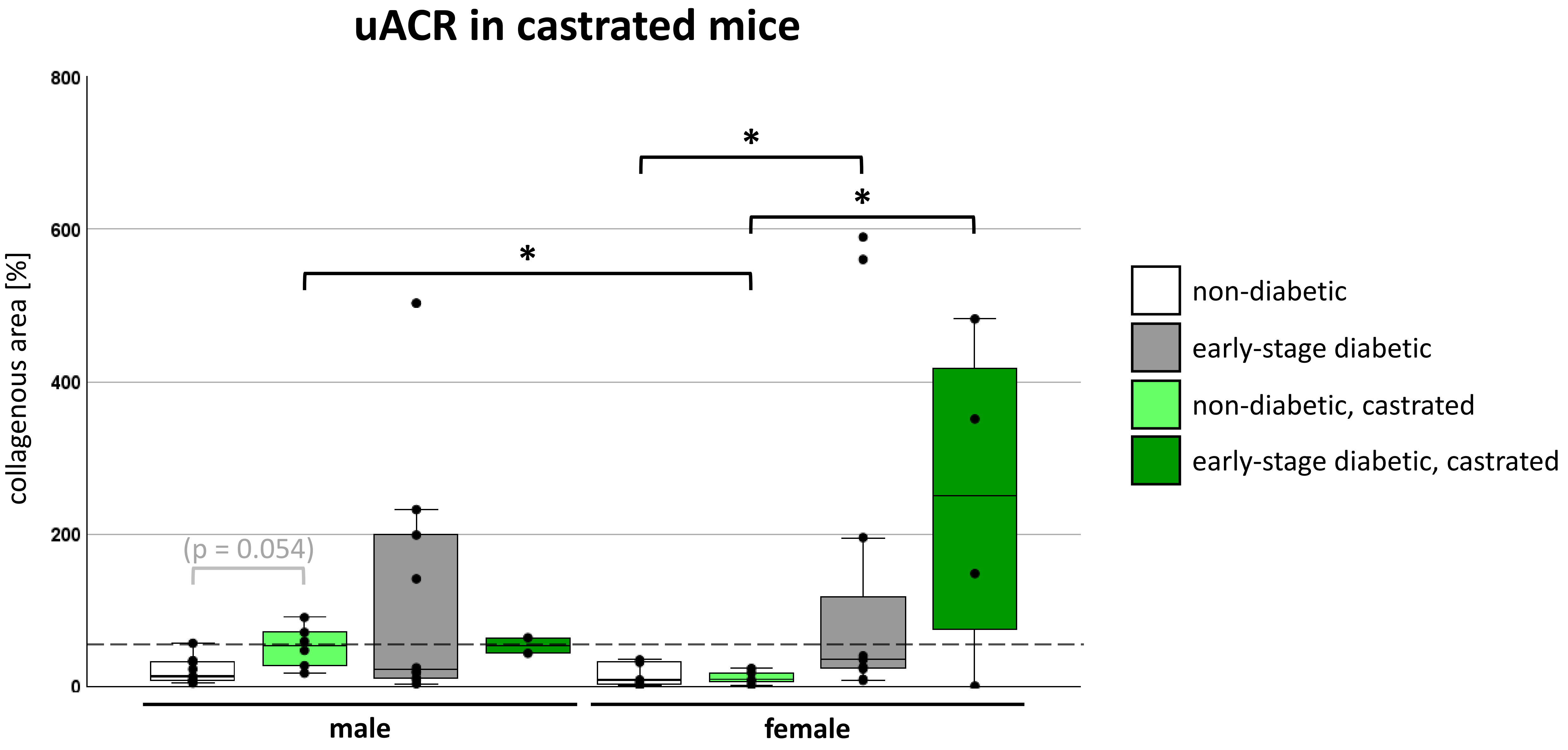

Supplementary Figure S2

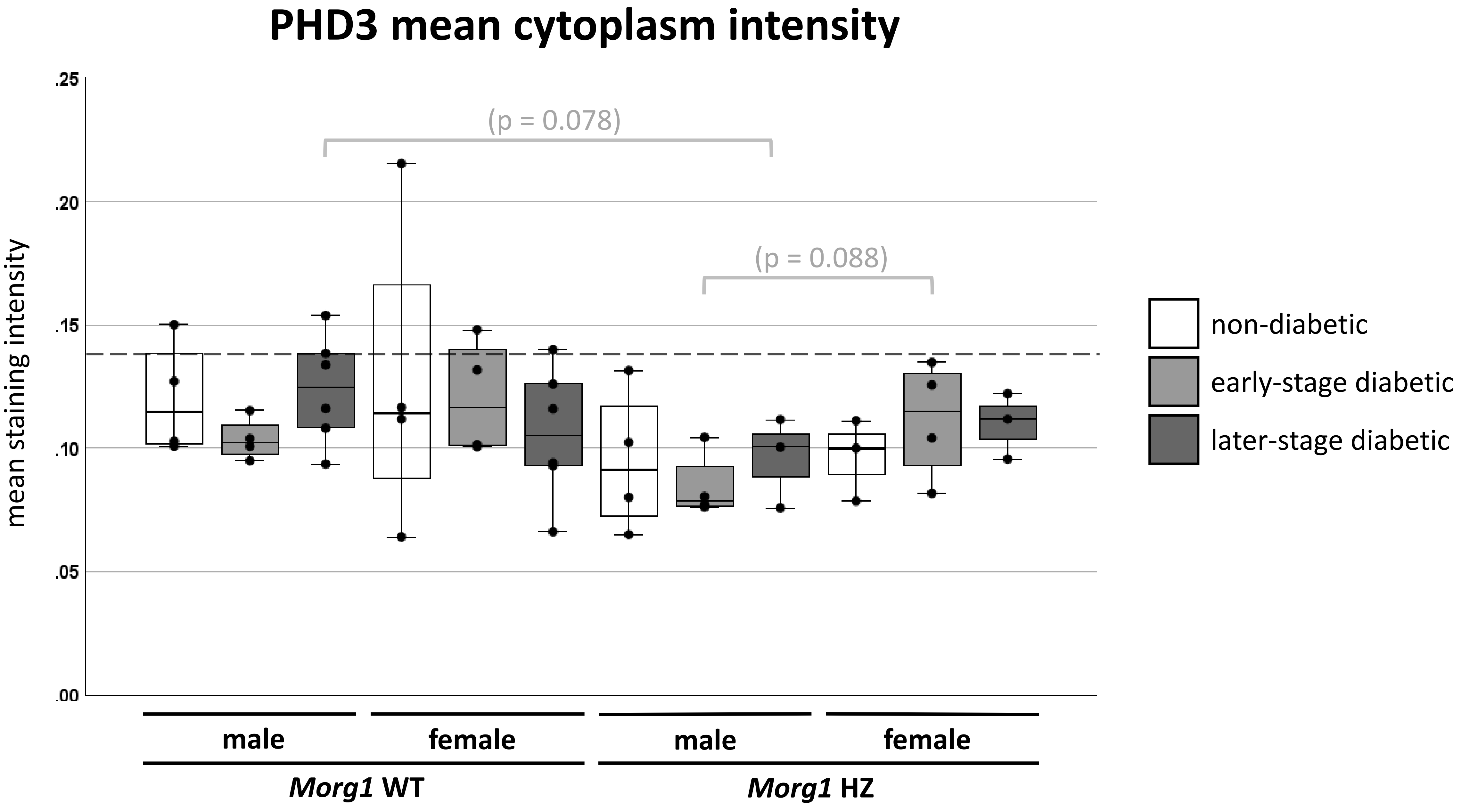

Supplementary Figure S3

Gene	Primer sequence	Tann	Reference
Hprt1	fwd 5`-TGGATACAGGCCAGACTTTGTT-3`<br>rev 5`-CAGATTCAACTTGCGCTCATC-3`	59°C	[19]
Gapdh	fwd 5`-CAATGACCCCTTCATTGACC-3`<br>rev 5`-TGGACTCCACGACGTACTCA-3`	59°C	[32]
Morg1	fwd 5`-CCTATCACCTGCACCTGCTT-3`<br>rev 5`-CACTTTCCCGTCTTCAGAGC-3`	58°C	[19]
Wdr83os	fwd 5`-GGCATGATCTTCAGCATGTGTGG-3`<br>rev 5`-ACATCACCACGGCGGAGATAGA-3`	62°C	in this study
Dhps	fwd 5`-CATACCTTGGCGAGTTCAGCCT-3`<br>rev 5`-CAGCACCATCTGGTCCAGAATG-3`	59°C	in this study

Supplementary Table S1

Antibody	Source	Dilution	Application
Mouse anti-ERK1 (monoclonal, clone unknown)	BD Biosciences (San Jose, CA, USA)	3:10.000	WB
Mouse anti-Phospho-p44/42 MAPK (ERK1/2) (T202/Y204) (monoclonal, clone E10)	Cell Signaling (Danvers, MA, USA)	1:2000	WB
Rabbit anti-p70S6 Kinase (polyclonal)	Cell Signaling (Danvers, MA, USA)	1:1000	WB
Rabbit anti-Phospho-p70S6 Kinase (T389) (polyclonal)	Cell Signaling (Danvers, MA, USA)	1:1000	WB
Mouse anti-Vinculin (monoclonal, clone hVIN-1)	Merck KGaG (Darmstadt, Germany)	1:1000	WB
Rabbit anti-HIF-1 alpha (polyclonal)	Novus Biologicals (Centennial, CO, USA)	1:250	IHC
Rabbit anti-EGLN3/PHD3 (polyclonal)	Thermo Fisher Scientific (Waltham, MA, USA)	1:200	IHC

Supplementary Table S2

Position	Antibody	Source	Antigen retrieval buffer	Antibody dilution	Opal™ reagent	Opal™ dilution
1	Rabbit anti-Synaptopodin (monoclonal, clone EPR23922-79)	Abcam, Cambridge, UK	AR9 (pH9)	1:500	480	1:100
2	Rabbit anti-Ggt1 (polyclonal)	Thermo Fisher Scientific, Waltham, MA, USA	AR6 (pH6)	1:2000	520	1:100
3	Rabbit anti-MORG1 (polyclonal)	Boster Bio, Pleasanton, CA, USA	AR9 (pH9)	1:200	570	1:100
4	Rabbit anti-DHPS (polyclonal)	Proteintech, Rosemont, IL, USA	AR9 (pH9)	1:100	620	1:100
5	Rabbit anti-WDR83os (polyclonal)	Thermo Fisher Scientific, Waltham, MA, USA	AR9 (pH9)	1:100	690	1:100
6	Rabbit anti-Sox9 (monoclonal, clone EPR14335)	Abcam, Cambridge, UK	AR9 (pH9)	1:2000	780	1:100
7					DAPI	

Supplementary Table S3

Renal fibrosis (Figure 1)

Factor	P-value
sex	**0.011***
diabetes	0.843
diabetes duration	**<0.001*****
Morg1 genotype	**<0.001*****
sex*diabetes	0.627
sex*diabetes duration	**0.007****
sex**Morg1* genotype	0.616
diabetes*diabetes duration	.
diabetes**Morg1* genotype	0.660
diabetes duration**Morg1* genotype	0.216
sex*diabetes*diabetes duration	.
sex*diabetes**Morg1* genotype	0.964
sex*diabetes duration**Morg1* genotype	0.975
diabetes*diabetes duration**Morg1* genotype	.
sex*diabetes*diabetes duration**Morg1* genotype	.

uACR (Figure 2)

Factor	P-value
sex	0.666
diabetes	**0.045***
diabetes duration	**<0.001*****
Morg1 genotype	0.957
sex*diabetes	0.938
sex*diabetes duration	0.975
sex**Morg1* genotype	0.711
diabetes*diabetes duration	.
diabetes**Morg1* genotype	**0.054(*)**
diabetes duration**Morg1* genotype	**0.081(*)**
sex*diabetes*diabetes duration	.
sex*diabetes**Morg1* genotype	0.238
sex*diabetes duration**Morg1* genotype	0.616
diabetes*diabetes duration**Morg1* genotype	.
sex*diabetes*diabetes duration**Morg1* genotype	.

HIF-1α positive nuclei (Figure 3A)

Factor	P-value
sex	0.897
diabetes	**0.066(*)**
diabetes duration	0.586
Morg1 genotype	0.142
sex*diabetes	0.716
sex*diabetes duration	0.272
sex**Morg1* genotype	**0.068(*)**
diabetes*diabetes duration	.
diabetes**Morg1* genotype	0.136
diabetes duration**Morg1* genotype	**0.073(*)**
sex*diabetes*diabetes duration	.
sex*diabetes**Morg1* genotype	**0.040***
sex*diabetes duration**Morg1* genotype	0.845
diabetes*diabetes duration**Morg1* genotype	.
sex*diabetes*diabetes duration**Morg1* genotype	.

HIF-1α cytoplasmic intensity (Figure 3B)

Factor	P-value
sex	0.414
diabetes	0.158
diabetes duration	0.226
Morg1 genotype	0.390
sex*diabetes	0.780
sex*diabetes duration	0.154
sex**Morg1* genotype	0.351
diabetes*diabetes duration	.
diabetes**Morg1* genotype	0.195
diabetes duration**Morg1* genotype	**0.019***
sex*diabetes*diabetes duration	.
sex*diabetes**Morg1* genotype	0.547
sex*diabetes duration**Morg1* genotype	0.360
diabetes*diabetes duration**Morg1* genotype	.
sex*diabetes*diabetes duration**Morg1* genotype	.

HIF-1α BBC-HS (Figure 3C)

Factor	P-value
sex	**<0.001*****
diabetes	**0.019***
diabetes duration	**0.046***
Morg1 genotype	0.524
sex*diabetes	0.485
sex*diabetes duration	**0.008****
sex**Morg1* genotype	0.902
diabetes*diabetes duration	.
diabetes**Morg1* genotype	0.688
diabetes duration**Morg1* genotype	**0.084(*)**
sex*diabetes*diabetes duration	.
sex*diabetes**Morg1* genotype	0.178
sex*diabetes duration**Morg1* genotype	0.469
diabetes*diabetes duration**Morg1* genotype	.
sex*diabetes*diabetes duration**Morg1* genotype	.

mTORC1 activity (Figure 4B)

Factor	P-value
sex	0.136
diabetes	0.780
diabetes duration	0.594
Morg1 genotype	0.798
sex*diabetes	0.960
sex*diabetes duration	0.863
sex**Morg1* genotype	0.665
diabetes*diabetes duration	.
diabetes**Morg1* genotype	**0.091(*)**
diabetes duration**Morg1* genotype	**<0.001*****
sex*diabetes*diabetes duration	.
sex*diabetes**Morg1* genotype	0.588
sex*diabetes duration**Morg1* genotype	0.527
diabetes*diabetes duration**Morg1* genotype	.
sex*diabetes*diabetes duration**Morg1* genotype	.

ERK1/2 activity (Figure 4C)

Factor	P-value
sex	0.753
diabetes	0.764
diabetes duration	**0.095(*)**
Morg1 genotype	**0.002****
sex*diabetes	0.596
sex*diabetes duration	0.298
sex**Morg1* genotype	0.830
diabetes*diabetes duration	.
diabetes**Morg1* genotype	0.343
diabetes duration**Morg1* genotype	0.222
sex*diabetes*diabetes duration	.
sex*diabetes**Morg1* genotype	0.844
sex*diabetes duration**Morg1* genotype	0.417
diabetes*diabetes duration**Morg1* genotype	.
sex*diabetes*diabetes duration**Morg1* genotype	.

Sox9 quantification (Figure 5B)

Factor	P-value
diabetes	**0.040***
celltype	0.848
diabetes*celltype	0.778

celltype quantification (Figure 5C)

Factor	P-value
diabetes	**0.040***
celltype	0.848
diabetes*celltype	0.778

Renal fibrosis in castrated mice (Supplementary Figure 1)

Factor	P-value
sex	0.639
diabetes	**0.018***
castration	0.451
sex*diabetes	0.354
sex*castration	0.835
diabetes*castration	**0.009****
sex*diabetes*castration	0.767

uACR in castrated mice (Supplementary Figure 2)

Factor	P-value
sex	0.328
diabetes	**0.007****
castration	0.335
sex*diabetes	0.127
sex*castration	0.559
diabetes*castration	0.806
sex*diabetes*castration	0.492

PHD3 cytoplasmic intensity (Supplementary Figure 3)

Factor	P-value
sex	0.536
diabetes	0.648
diabetes duration	0.695
Morg1 genotype	**0.031***
sex*diabetes	0.380
sex*diabetes duration	0.224
sex**Morg1* genotype	0.446
diabetes*diabetes duration	.
diabetes**Morg1* genotype	0.488
diabetes duration**Morg1* genotype	0.917
sex*diabetes*diabetes duration	.
sex*diabetes**Morg1* genotype	0.704
sex*diabetes duration**Morg1* genotype	0.579
diabetes*diabetes duration**Morg1* genotype	.
sex*diabetes*diabetes duration**Morg1* genotype	.

For each outcome, the corresponding figure panel is indicated. Significant effects and statistical trends are highlighted in bold.

Supplementary Table S4

	Morg1 mRNA expression	Wdr83os mRNA expression	Dhps mRNA expression	Renal fibrosis	Albuminuria (uACR)	ERK1/2 activity	mTORC1 activity (p70S6K)	HIF-1a expression	PHD3 expression
**Morg1 mRNA expression**	122	122	122	122	63	56	55	52	49
**Wdr83os mRNA expression**	122	122	122	122	63	56	55	52	49
**Dhps mRNA expression**	122	122	122	122	63	56	55	52	49
**Renal fibrosis**	122	122	122	122	63	56	55	52	49
**Albuminuria (uACR)**	63	63	63	63	101	37	36	33	32
**ERK1/2 activity**	56	56	56	56	37	56	55	52	49
**mTORC1 activity (p70S6K)**	55	55	55	55	36	55	55	51	48
**HIF-1a expression**	52	52	52	52	33	52	51	52	46
**PHD3 expression**	49	49	49	49	32	49	48	46	49

Supplementary Table S5

Main table	Outcome	Comparison	*p* value
Table 1	Weight	female non-diabetic vs. early-stage diabetic *Morg1* WT	<0.001
Table 1	Weight	female non-diabetic vs. later-stage diabetic *Morg1* HZ	<0.001
Table 1	Weight	female non-diabetic vs. later-stage diabetic *Morg1* WT	<0.001
Table 1	Weight	female non-diabetic vs. early-stage diabetic *Morg1* HZ	<0.001
Table 1	Weight	male non-diabetic vs. later-stage diabetic *Morg1* WT	<0.001
Table 1	Weight	male non-diabetic vs. later-stage diabetic *Morg1* HZ	0.001
Table 1	Weight	ovariectomized female non-diabetic vs. early-stage diabetic *Morg1* WT	0.007
Table 1	Weight	male non-diabetic vs. early-stage diabetic *Morg1* WT	0.022
Table 1	Weight	male vs. female non-diabetic *Morg1* HZ	0.045
Table 1	Weight	male vs. female non-diabetic *Morg1* WT	0.079
Table 1	Weight	male non-diabetic vs. early-stage diabetic *Morg1* HZ	0.083
Table 1	Weight	male early-stage vs. later-stage diabetic *Morg1* HZ	0.085
Table 1	Weight	orchiectomized male non-diabetic vs. early-stage diabetic *Morg1* WT	0.095
Table 1	Fasting blood glucose	male non-diabetic vs. later-stage diabetic *Morg1* WT	<0.001
Table 1	Fasting blood glucose	orchiectomized male non-diabetic vs. early-stage diabetic *Morg1* WT	0.001
Table 1	Fasting blood glucose	male non-diabetic vs. early-stage diabetic *Morg1* WT	0.003
Table 1	Fasting blood glucose	female non-diabetic vs. early-stage diabetic *Morg1* WT	0.008
Table 1	Fasting blood glucose	female non-diabetic vs. later-stage diabetic *Morg1* WT	0.016
Table 1	Fasting blood glucose	female early-stage diabetic *Morg1* WT vs. HZ	0.026
Table 2	*Morg1* mRNA	female early-stage diabetic *Morg1* WT vs. HZ	<0.001
Table 2	*Morg1* mRNA	female non-diabetic *Morg1* WT vs. HZ	<0.001
Table 2	*Morg1* mRNA	male later-stage diabetic *Morg1* WT vs. HZ	0.001
Table 2	*Morg1* mRNA	male early-stage diabetic *Morg1* WT vs. HZ	0.009
Table 2	*Morg1* mRNA	female later-stage diabetic *Morg1* WT vs. HZ	0.012
Table 2	*Wdr83os* mRNA	female vs. ovariectomized female early-stage diabetic *Morg1* WT	0.019
Table 2	*Wdr83os* mRNA	ovariectomized female non-diabetic vs. early-stage diabetic *Morg1* WT	0.026
Table 2	*Wdr83os* mRNA	male vs. orchiectomized male non-diabetic *Morg1* WT	0.034
Table 2	*Wdr83os* mRNA	orchiectomized male non-diabetic vs. early-stage diabetic *Morg1* WT	0.061
Table 2	*Wdr83os* mRNA	female later-stage diabetic *Morg1* WT vs. HZ	0.062
Table 2	*Wdr83os* mRNA	female non-diabetic vs. later-stage diabetic *Morg1* HZ	0.063
Table 2	*Wdr83os* mRNA	male non-diabetic vs. later-stage diabetic *Morg1* HZ	0.075
Table 2	*Dhps* mRNA	male vs. orchiectomized male early-stage diabetic *Morg1* WT	0.003
Table 2	*Dhps* mRNA	orchiectomized male non-diabetic vs. early-stage diabetic *Morg1* WT	0.011
Table 2	*Dhps* mRNA	male non-diabetic vs. early-stage diabetic *Morg1* WT	0.016
Table 2	*Dhps* mRNA	female early-stage diabetic *Morg1* WT vs. HZ	0.032
Table 2	*Dhps* mRNA	male vs. orchiectomized male non-diabetic *Morg1* WT	0.075

Supplementary Table S6
