# Supplementary material for: MORG1 Haploinsufficiency Is Associated with Stage- and Sex-Dependent Renoprotection and Signalling Alterations in Diabetic Kidney Disease
Source: Int J Mol Sci. 2026 Sep 8;27(18):7990. doi: 10.3390/ijms27187990 (PMC13606655; doi:10.3390/ijms27187990)
Supplement: Supplementary file 1 [file ijms-27-07990-s001.zip › Supplementary Methods.pdf]

Supplementary Methods

Animals

All animal experiments were approved by the local ethics committee (UKJ-17-024, UKJ-22-026, 02-023-16, UKJ-22-008) and performed in accordance with German animal protection law. The mouse line was generated by crossing *db/db* mice (B6.Cg-Dock7m Leprdb/++/J; Charles River) with an *Morg1* knockout strain as previously described.[19] Leptin receptor wild-type mice served as non-diabetic controls, whereas homozygous leptin receptor mutant mice were classified as diabetic. Homozygous *Morg1* knockout mice are embryonically lethal; therefore, only heterozygous *Morg1* knockout mice have been studied.[25]

The animals were housed under specific pathogen-free conditions with a 12 h light/dark cycle and free access to food and water. All animals were housed and handled under identical conditions to minimize potential confounding factors. Randomization was not applicable because the animals were allocated to experimental groups according to their predefined *Morg1* genotype (wild-type (WT) or heterozygous (HZ)), sex (male or female), diabetic status (non-diabetic or diabetic), and disease stage (early stage and later stage). For sex hormone studies, male and female *Morg1* wild-type mice (non-diabetic and early-stage diabetic) were subjected to orchiectomy or ovariectomy at 6–8 weeks of age. At the end of the experiment, the age of the animals was as follows: male non-diabetic *Morg1* WT, 25.33 ± 2.26 weeks; male non-diabetic *Morg1* HZ, 24.86 ± 3.61 weeks; male early-stage diabetic *Morg1* WT, 23.83 ± 3.76 weeks; male early-stage diabetic *Morg1* HZ, 23.50 ± 4.12 weeks; male later-stage diabetic *Morg1* WT, 61.00 ± 15.90 weeks; male later-stage diabetic *Morg1* HZ, 62.17 ± 16.73 weeks; male (orchiectomized) non-diabetic *Morg1* WT, 25.38 ± 5.58 weeks; male (orchiectomized) early-stage diabetic *Morg1* WT, 27.00 ± 2.45 weeks; female non-diabetic *Morg1* WT, 25.70 ± 1.49 weeks; female non-diabetic *Morg1* HZ, 23.36 ± 3.53 weeks; female early-stage diabetic *Morg1* WT, 25.11 ± 1.94 weeks; female early-stage diabetic *Morg1* HZ, 24.90 ± 3.07 weeks; female later-stage diabetic *Morg1* WT, 53.25 ± 15.25 weeks; female later-stage diabetic *Morg1* HZ, 59.60 ± 21.65 weeks; female (ovariectomized) non-diabetic *Morg1* WT, 25.73 ± 7.24 weeks; and female (ovariectomized) early-stage diabetic *Morg1* WT, 25.50 ± 3.70 weeks. The experimental unit was the individual animal. Depending on the analysis performed, the group sizes ranged from $n = 4$ to $n = 12$ mice, with a total of 122 animals included in the study. The sample size was determined via an a priori power analysis ($\alpha = 0.05$, power = 80%) for the primary fibrosis endpoint in male mice. The analysis indicated that 8 animals per group were needed for the comparison between early- and late-stage diabetic WT mice, and 6 animals per group were needed for the corresponding comparison of diabetic *Morg1* heterozygous mice. Female cohorts were included to investigate sex-dependent effects. No predefined exclusion criteria for animals were applied. The number of animals available for individual analyses varied because not all biological samples (e.g., urine or kidney tissue) were available from every animal and because some analyses were performed in independent or partially overlapping cohorts. The exact number of animals per group is reported in the corresponding figure legends and tables. No inclusion or exclusion criteria were set.

Twenty-four-hour urine samples were collected in metabolic cages before sacrifice. Blood and kidneys were harvested; one kidney half was fixed in 4% formalin for histology, and the remaining tissue was snap-frozen for RNA and protein analyses.

Digital Azan staining analysis

Whole-slide images were acquired with a Vectra Polaris™ scanner (Akoya Biosciences, now Quanterix). Collagen-positive areas were quantified via pixel-based image analysis in QuPath version 0.5.1.[26] The staining vectors were optimized for each section, and the results are presented as percentages of the annotated area. Kidney sections were labelled only with the individual animal identification number. Azan staining, image acquisition, and quantitative analysis were performed by investigators who were blinded to genotype, diabetic status, sex, and disease stage.

Digital immunohistochemistry analysis

Whole-cortex regions were analysed in QuPath version 0.7.0.[26] The mean cytoplasmic staining intensity, percentage of positive nuclei, and blue–brown color H score were quantified via custom Groovy scripts.[27] Scripts are provided in the supplementary information. Kidney sections were labelled only with the individual animal identification number. Immunohistochemical staining, image acquisition, and quantitative analysis were performed by investigators who were blinded to the genotype, diabetic status, sex, and disease stage of the patients.

Multiplex immunofluorescence

Multiplex immunofluorescence staining was performed via the Opal™ 3-Plex Anti-Rb Detection Kit (Akoya Biosciences, now Quanterix) according to the manufacturer's instructions supplemented with additional Opal™ reagents.[27] The target antigens were Synaptopodin, GGT1, MORG1, DHPS, WDR83os, and SOX9, with DAPI nuclear counterstaining. The staining conditions were optimized against the corresponding monoplex staining conditions and are summarized in **Suppl**. **Table 3**.

Whole-slide images were acquired via a Vectra Polaris™ scanner. Digital image analysis was performed in QuPath version 0.7.0.[26] The cells were classified as podocytes, proximal tubular cells, or others via a random trees classifier. Marker expression was quantified via a custom Groovy script provided in the supplementary information.

Detailed statistical analysis

The outcome measures assessed in this study included body weight, fasting blood glucose, the urinary albumin-to-creatinine ratio, renal fibrosis (Azan staining), HIF-1α immunohistochemistry, mTORC1 and ERK signalling (Western blot), and renal gene expression (RT–qPCR). The primary outcome measure used for sample size calculation was renal fibrosis assessed by Azan staining in diabetic male mice. All other outcome measures were secondary analyses. Data are presented as box-and-whisker plots with individual data points; boxes indicate the median and interquartile range, and whiskers extend to the most extreme values within 1.5 × IQR. No animals or data points were excluded from the analyses after allocation to the experimental groups. Differences in sample size between analyses reflect biological sample availability (e.g., urine or kidney tissue) and the use of independent or partially overlapping cohorts for specific outcome measures rather than post hoc exclusion of animals or data. The exact sample size (n) for each analysis is indicated in the corresponding figure legends and tables. Statistical analyses were performed via IBM SPSS Statistics (IBM, Armonk, NY, USA). Multiway ANOVA was used to assess the main effects and interactions of sex, diabetes, genotype, and disease stage. Model assumptions were evaluated via the Shapiro–Wilk test, Q–Q plots, and Levene's test. Variables with a skewed distribution (renal fibrosis, urinary albumin-to-creatinine ratio, and ERK1/2 activity) were log10-transformed before analysis. Planned pairwise comparisons between individual groups were performed via Mann–Whitney U tests. Because of the exploratory nature of the study, no correction for multiple testing was applied. Statistical significance was defined as $p \leq 0.05$, and p values between 0.05 and 0.10 were considered indicative of a trend.

Supplementary Figure and Table legends

Figure S1: Analysis of renal fibrosis by Azan trichrome staining in castrated mice. The quantification of collagen blue areas is shown as a percentage of the whole cortical area. Significance levels: * $p < 0.05$, ** $p < 0.01$, *** $p < 0.001$. *N = 11/8/10/4/8/8/12/4 (groups from left to right).*

Figure S2: Analysis of the urinary albumin-to-creatinine ratio (uACR) in castrated animals. Normoalbuminuria: <30 mg/g creatinine; microalbuminuria: 30–300 mg/g creatinine; macroalbuminuria: >300 mg/g creatinine. Significance levels: * $p < 0.05$, ** $p < 0.01$, *** $p < 0.001$. *N = 11/6/10/2/8/5/11/4 (groups from left to right).*

Figure S3: Analysis of PHD3 protein expression, quantified as the mean cytoplasmic intensity. Significance levels: * $p < 0.05$, ** $p < 0.01$, *** $p < 0.001$; gray (*) indicates statistical trends ($p < 0.1$). *N = 4/4/6/4/4/6/4/4/3/3/4/3 (groups from left to right).*

Table S1: Primer sets and their respective annealing temperatures for genes analysed by real-time PCR (provided by TIB MOLBIOL Syntheselabor GmbH (Berlin, Germany)).

Tann: Annealing temperature, Hprt1: hypoxanthine phosphoribosyltransferase 1, Gapdh: glyceraldehyde 3-phosphate dehydrogenase, Morg1: mitogen-activated protein kinase organizer 1, Wdr83os: WDR83 opposite strand, Dhps: deoxyhypusine synthase, fwd: forward primer, rev: reverse primer.

Table S2: Antibodies used for western blotting and immunohistochemistry.

ERK1: extracellular signal-regulated kinase 1, MAPK: mitogen-activated protein kinase, WB: western blot, IHC: immunohistochemistry.

Table S3: Overview of multiplex immunofluorescence staining parameters and sequences. The table summarizes the sequential staining order (positions 1--6), antibody, source, antigen retrieval conditions, antibody dilution, assigned fluorophore, and Opal reagent dilution. Final (position 7) nuclear counterstaining was performed with DAPI.

AR: antigen retrieval, GGT1: gamma-glutamyl transferase 1, MORG1: mitogen-activated protein kinase organizer 1, DHPS: deoxyhypusine synthase, WDR83os: WDR83 opposite strand, SOX9: SRY-related HMG-box 9, DAPI: 4′,6-diamidino-2-phenylindole

Table S4: Summary of the results of the multiway ANOVA for all the quantitative readouts.

Table S5: Overlap of animal cohorts across experimental readouts. The numbers indicate the number of individual animals contributing to both experimental readouts indicated by the corresponding row and column. The diagonal values represent the total number of animals included in each respective analysis.

Table S6: Exact p values for the planned pairwise comparisons corresponding to the statistical annotations in Tables 1 and 2. Pairwise comparisons were performed via Mann–Whitney U tests as described in the Statistical Analysis section. Comparisons of the effects of sex, diabetes status, disease stage, genotype, and gonadectomy within the corresponding experimental groups were performed. P values are reported without adjustment for multiple testing because these secondary analyses were exploratory. WT: wild-type; HZ: heterozygous

Groovy script for analysis of HIF-1α protein expression expression in immunohistochemistry via QuPath; positive nuclei and mean cytoplasm intensity (Figure 3A-B)

```
double nucleusThreshold = 0.4
double cellThreshold    = 0.4

String nucleusThresholdMeasurement = "Nucleus: DAB OD mean"
String cellThresholdMeasurement    = "Cytoplasm: DAB OD mean"

String nucleusMeanMeasurement = "Nucleus: DAB OD mean"
String cellMeanMeasurement    = "Cytoplasm: DAB OD mean"

String outputName = "HIF_per_annotation_results.csv"

boolean meanOnlyPositive = false


import qupath.lib.gui.scripting.QPEx
import java.nio.file.Files
import java.nio.file.Paths
import java.nio.charset.StandardCharsets


def project = QPEx.getProject()
if (project == null) {
    print "No project open."
    return
}

def outputPath = Paths.get(QPEx.buildFilePath(QPEx.PROJECT_BASE_DIR, outputName))

def lines = []
lines << [
    "Image Name",
    "Annotation size",
    "# Detections",
    "Hif positive nuclei (over threshold)",
    "Mean intensity Hif nuclei",
    "Hif positive cells (over threshold)",
    "Mean intensity Hif cells"
].join(",")


for (entry in project.getImageList()) {

    print "Processing image: ${entry.getImageName()}"

    def imageData = entry.readImageData()
    def hierarchy = imageData.getHierarchy()

    def annotations = hierarchy.getAnnotationObjects()
    def cells = hierarchy.getCellObjects()

    if (annotations == null || annotations.isEmpty()) {
        print "  No annotations found."
        continue
    }
```

```
    if (cells == null || cells.isEmpty()) {
        print "  No cell detections found."
        continue
    }

    for (ann in annotations) {

        def annROI = ann.getROI()
        if (annROI == null)
            continue

        def cellsInAnnotation = cells.findAll { cell ->
            def roi = cell.getROI()
            roi != null && annROI.contains(roi.getCentroidX(), roi.getCentroidY())
        }

        int nDetections = cellsInAnnotation.size()

        int positiveNuclei = 0
        int positiveCells  = 0

        double nucleusSum = 0
        int nucleusCount = 0

        double cellSum = 0
        int cellCount = 0

        for (cell in cellsInAnnotation) {
            def ml = cell.getMeasurementList()

            double nucThreshVal = ml.containsKey(nucleusThresholdMeasurement) ?
ml.get(nucleusThresholdMeasurement) : Double.NaN
            double cellThreshVal = ml.containsKey(cellThresholdMeasurement) ?
ml.get(cellThresholdMeasurement) : Double.NaN

            double nucMeanVal = ml.containsKey(nucleusMeanMeasurement) ? ml.get(nucleusMeanMeasurement)
: Double.NaN
            double cellMeanVal = ml.containsKey(cellMeanMeasurement) ? ml.get(cellMeanMeasurement) :
Double.NaN

            boolean nucPositive = !Double.isNaN(nucThreshVal) && nucThreshVal > nucleusThreshold
            boolean cellPositive = !Double.isNaN(cellThreshVal) && cellThreshVal > cellThreshold

            if (nucPositive)
                positiveNuclei++

            if (cellPositive)
                positiveCells++

            if (!Double.isNaN(nucMeanVal)) {
                if (meanOnlyPositive) {
                    if (nucPositive) {
                        nucleusSum += nucMeanVal
                        nucleusCount++
                    }
                } else {
                    nucleusSum += nucMeanVal
```

```
                nucleusCount++
            }
        }

        if (!Double.isNaN(cellMeanVal)) {
            if (meanOnlyPositive) {
                if (cellPositive) {
                    cellSum += cellMeanVal
                    cellCount++
                }
            } else {
                cellSum += cellMeanVal
                cellCount++
            }
        }
    }

    double meanNucleus = nucleusCount > 0 ? nucleusSum / nucleusCount : Double.NaN
    double meanCell    = cellCount > 0 ? cellSum / cellCount : Double.NaN

    double annArea = Double.NaN
    try {
        annArea = annROI.getArea()
    } catch (Exception e) {
        annArea = Double.NaN
    }

    lines << [
        "\"${entry.getImageName()}\"",
        annArea,
        nDetections,
        positiveNuclei,
        meanNucleus,
        positiveCells,
        meanCell
    ].join(",")
  }

  entry.saveImageData(imageData)
}


Files.write(outputPath, lines, StandardCharsets.UTF_8)

print "Done."
print "CSV saved to: ${outputPath}"
```

Groovy script for analysis of HIF-1α protein expression expression in immunohistochemistry via QuPath; BBC-HS (Figure 3C)

```
String measurement = "Nucleus: DAB OD max"

double c1 = 0.1
double c2 = 0.2
double c3 = 0.4
```

```
String outputName = "HIF_Hscore_per_image.csv"


import qupath.lib.gui.scripting.QPEx
import java.nio.file.Files
import java.nio.file.Paths
import java.nio.charset.StandardCharsets
import java.util.Locale


def project = QPEx.getProject()
if (project == null) {
  print "No project open."
  return
}

def outputPath = Paths.get(QPEx.buildFilePath(QPEx.PROJECT_BASE_DIR, outputName))

def lines = []
lines << [
  "Image Name",
  "Total cells",
  "% 1+",
  "% 2+",
  "% 3+",
  "H-Score"
].join(";")


for (entry in project.getImageList()) {

  print "Processing: ${entry.getImageName()}"

  def imageData = null
  try {
    imageData = entry.readImageData()
  } catch (Exception e) {
    print "  ERROR reading image data for ${entry.getImageName()}: ${e.getMessage()}"
    continue
  }

  def cells = imageData.getHierarchy().getCellObjects()

  // Include images with no cells
  if (cells == null || cells.isEmpty()) {
    lines << [
      "\"${entry.getImageName()}\"",
      0,
      "0,00",
      "0,00",
      "0,00",
      "0,00"
    ].join(";")
    print "  No cells found -> writing zeros"
    continue
  }
```

```
int n0 = 0
int n1 = 0
int n2 = 0
int n3 = 0
int valid = 0

for (cell in cells) {
    def ml = cell.getMeasurementList()

    if (!ml.containsKey(measurement))
        continue

    double val = ml.get(measurement)
    if (Double.isNaN(val))
        continue

    valid++

    if (val < c1) {
        n0++
    } else if (val < c2) {
        n1++
    } else if (val < c3) {
        n2++
    } else {
        n3++
    }
}

if (valid == 0) {
    lines << [
        "\"${entry.getImageName()}\"",
        cells.size(),
        "0,00",
        "0,00",
        "0,00",
        "0,00"
    ].join(";")
    print "  No valid measurements -> writing zeros"
    continue
}

double p1 = ((double)n1 / (double)valid) * 100.0
double p2 = ((double)n2 / (double)valid) * 100.0
double p3 = ((double)n3 / (double)valid) * 100.0

double hscore = (1.0 * p1) + (2.0 * p2) + (3.0 * p3)

String p1s = String.format(Locale.GERMAN, "%.2f", p1)
String p2s = String.format(Locale.GERMAN, "%.2f", p2)
String p3s = String.format(Locale.GERMAN, "%.2f", p3)
String hs  = String.format(Locale.GERMAN, "%.2f", hscore)

lines << [
    "\"${entry.getImageName()}\"",
    valid,
    p1s,
    p2s,
```

```
        p3s,
        hs
    ].join(";")
}


Files.write(outputPath, lines, StandardCharsets.UTF_8)

print "Done."
print "Saved to: ${outputPath}"
```

Groovy script for analysis of PHD3 protein expression in immunohistochemistry via QuPath; mean cytoplasm intensity (Supplementary Figure 3)

```
String cytoplasmMeanMeasurement = "Cytoplasm: DAB OD mean"
String outputName = "PHD3_cytoplasm_mean_per_annotation.csv"


import qupath.lib.gui.scripting.QPEx
import java.nio.file.Files
import java.nio.file.Paths
import java.nio.charset.StandardCharsets


def project = QPEx.getProject()
if (project == null) {
   print "No project open."
   return
}

def outputPath = Paths.get(QPEx.buildFilePath(QPEx.PROJECT_BASE_DIR, outputName))

def lines = []
lines << [
   "Image Name",
   "Annotation size",
   "# Detections",
   "Mean cytoplasmic PHD3 intensity"
].join(",")


for (entry in project.getImageList()) {

   print "Processing image: ${entry.getImageName()}"

   def imageData = entry.readImageData()
   def hierarchy = imageData.getHierarchy()

   def annotations = hierarchy.getAnnotationObjects()
   def cells = hierarchy.getCellObjects()

   if (annotations == null || annotations.isEmpty()) {
      print "  No annotations found."
      continue
   }

   if (cells == null || cells.isEmpty()) {
      print "  No cell detections found."
      continue
   }

   for (ann in annotations) {

      def annROI = ann.getROI()
      if (annROI == null)
         continue
```

```
        def cellsInAnnotation = cells.findAll { cell ->
            def roi = cell.getROI()
            roi != null && annROI.contains(roi.getCentroidX(), roi.getCentroidY())
        }

        int nDetections = cellsInAnnotation.size()

        double cytoplasmSum = 0
        int cytoplasmCount = 0

        for (cell in cellsInAnnotation) {
            def ml = cell.getMeasurementList()

            double cytoplasmVal = ml.containsKey(cytoplasmMeanMeasurement) ?
                    ml.get(cytoplasmMeanMeasurement) : Double.NaN

            if (!Double.isNaN(cytoplasmVal)) {
                cytoplasmSum += cytoplasmVal
                cytoplasmCount++
            }
        }

        double meanCytoplasm = cytoplasmCount > 0 ?
                cytoplasmSum / cytoplasmCount : Double.NaN

        double annArea = Double.NaN
        try {
            annArea = annROI.getArea()
        } catch (Exception e) {
            annArea = Double.NaN
        }

        lines << [
            "\"${entry.getImageName()}\"",
            annArea,
            nDetections,
            meanCytoplasm
        ].join(",")
    }

    entry.saveImageData(imageData)
}


Files.write(outputPath, lines, StandardCharsets.UTF_8)

print "Done."
print "CSV saved to: ${outputPath}"
```

Groovy script for analysis of exploratory multiplex immunofluorescence via QuPath (Figure 5B-C)

```
import qupath.lib.common.GeneralTools
import qupath.lib.projects.Projects
import java.util.Locale

double thresholdMorg1   = 80.0
double thresholdDhps    = 65.0
double thresholdWdr83os = 50.0
double thresholdSox9    = 18.0


def options = [
   morg1  : ["Cell: MORG1 mean",    "Cell: Opal 570 mean"],
   dhps   : ["Cell: DHPS mean",     "Cell: Opal 620 mean"],
   wdr83os: ["Cell: WDR83os mean",  "Cell: Opal 690 mean"],
   sox9   : ["Nucleus: Sox9 max", "Nucleus: Opal 780 max"]
]


def fmt = { v ->
   if (v == null || Double.isNaN(v as double)) return ""
   return String.format(Locale.US, "%.4f", v as double)
}

def pct = { num, den ->
   if (den == null || den == 0) return Double.NaN
   return (100.0 * num) / den
}

def mean = { sum, n ->
   if (n == null || n == 0) return Double.NaN
   return sum / n
}

def getVal = { ml, key ->
   if (ml == null || key == null) return Double.NaN
   if (!ml.containsKey(key)) return Double.NaN
   def v = ml.get(key)
   return v == null ? Double.NaN : (v as double)
}

def newStats = {
   return [
      total              : 0,

      morg1_pos           : 0,
      dhps_pos           : 0,
      wdr83os_pos         : 0,
      sox9_pos           : 0,

      morg1_dhps          : 0,
      morg1_wdr83os       : 0,
      dhps_wdr83os        : 0,
      triple_mdw          : 0,
      quadruple           : 0,
```

```
        sox9_morg1          : 0,
        sox9_dhps           : 0,
        sox9_wdr83os        : 0,

        sum_morg1           : 0.0,
        sum_dhps            : 0.0,
        sum_wdr83os         : 0.0,
        sum_sox9            : 0.0,

        sum_morg1_in_sox9    : 0.0,
        sum_dhps_in_sox9     : 0.0,
        sum_wdr83os_in_sox9  : 0.0,
        sum_sox9_in_sox9     : 0.0
    ]
}

def updateStats = { stats, vMorg1, vDhps, vWdr83os, vSox9, isMorg1, isDhps, isWdr83os, isSox9 ->
    stats.total++
    stats.sum_morg1   += vMorg1
    stats.sum_dhps    += vDhps
    stats.sum_wdr83os += vWdr83os
    stats.sum_sox9    += vSox9

    if (isMorg1)   stats.morg1_pos++
    if (isDhps)    stats.dhps_pos++
    if (isWdr83os) stats.wdr83os_pos++
    if (isSox9)    stats.sox9_pos++

    if (isMorg1 && isDhps)    stats.morg1_dhps++
    if (isMorg1 && isWdr83os) stats.morg1_wdr83os++
    if (isDhps  && isWdr83os) stats.dhps_wdr83os++

    if (isMorg1 && isDhps && isWdr83os)
        stats.triple_mdw++

    if (isMorg1 && isDhps && isWdr83os && isSox9)
        stats.quadruple++

    if (isSox9) {
        stats.sum_morg1_in_sox9   += vMorg1
        stats.sum_dhps_in_sox9    += vDhps
        stats.sum_wdr83os_in_sox9 += vWdr83os
        stats.sum_sox9_in_sox9    += vSox9

        if (isMorg1)   stats.sox9_morg1++
        if (isDhps)    stats.sox9_dhps++
        if (isWdr83os) stats.sox9_wdr83os++
    }
}



def project = getProject()
if (project == null) {
    print "Dieses Skript bitte im Projektmodus mit 'Run for Project' ausführen.\n"
    return
}
```

```
def baseDir = Projects.getBaseDirectory(project)
def outCsv = new File(baseDir, "mif_celltype_summary.csv")

if (!outCsv.exists()) {
    outCsv.text =
        "image,group,total," +
        "mean_morg1_signal,mean_dhps_signal,mean_wdr83os_signal,mean_nuclear_sox9_signal," +
        "sox9_pos,sox9_pct," +
        "morg1_pos,morg1_pct," +
        "dhps_pos,dhps_pct," +
        "wdr83os_pos,wdr83os_pct," +
        "morg1_dhps,morg1_wdr83os,dhps_wdr83os," +
        "triple_mdw,triple_mdw_pct_of_all," +
        "quadruple,quadruple_pct_of_all,quadruple_pct_of_sox9," +
        "sox9_morg1,sox9_morg1_pct_of_sox9," +
        "sox9_dhps,sox9_dhps_pct_of_sox9," +
        "sox9_wdr83os,sox9_wdr83os_pct_of_sox9," +

"mean_morg1_signal_in_sox9,mean_dhps_signal_in_sox9,mean_wdr83os_signal_in_sox9,mean_nuclear_sox9
_signal_in_sox9\n"
}



def imageData = getCurrentImageData()
if (imageData == null) {
    print "Kein Bild geladen – wird übersprungen.\n"
    return
}

def server = imageData.getServer()
def imgName = GeneralTools.stripExtension(server.getMetadata().getName())

print "\n=== Verarbeite: ${imgName} ==="



def cells = getCellObjects()
if (cells == null || cells.isEmpty()) {
    print "No cells detected\n"
    ["Podocytes", "PT", "Other", "Unclassified", "All"].each { group ->
        outCsv << "${imgName},${group},0,,,,,0,,0,,0,,0,,0,0,0,0,,0,,0,,0,,0,,,,\n"
    }
    return
}

def example = cells.find { it.getMeasurementList() != null }
if (example == null) {
    print "Keine verwertbare Beispielzelle gefunden.\n"
    return
}

def meas = example.getMeasurementList()

def keys = [:]
options.each { marker, candidates ->
    def found = candidates.find { meas.containsKey(it) }
    keys[marker] = found
```

```
    if (found == null)
        print "No measurement found for ${marker}: ${candidates}\n"
}

def missing = keys.findAll { k, v -> v == null }.collect { it.key }
if (!missing.isEmpty()) {
    print "Fehlende Messkanäle: ${missing}\n"
    print "Skript abgebrochen – bitte Measurement-Namen prüfen.\n"
    return
}

print "Verwendete Messschlüssel:"
keys.each { k, v -> print "  ${k}: ${v}" }


def stats = [
    Podocytes   : newStats(),
    PT          : newStats(),
    Other       : newStats(),
    Unclassified: newStats(),
    All         : newStats()
]


for (cell in cells) {

    def mm = cell.getMeasurementList()
    if (mm == null) continue

    def vMorg1   = getVal(mm, keys.morg1)
    def vDhps    = getVal(mm, keys.dhps)
    def vWdr83os = getVal(mm, keys.wdr83os)
    def vSox9    = getVal(mm, keys.sox9)

    if ([vMorg1, vDhps, vWdr83os, vSox9].any { Double.isNaN(it as double) })
        continue

    def isMorg1   = vMorg1   > thresholdMorg1
    def isDhps    = vDhps    > thresholdDhps
    def isWdr83os = vWdr83os > thresholdWdr83os
    def isSox9    = vSox9    > thresholdSox9

    def cls = cell.getPathClass()?.toString() ?: ""
    def group = "Unclassified"

    if (cls.contains("Podocytes")) {
        group = "Podocytes"
    } else if (cls.contains("PT")) {
        group = "PT"
    } else if (cls.contains("Other")) {
        group = "Other"
    } else {
        group = "Unclassified"
    }

    updateStats(stats[group], vMorg1, vDhps, vWdr83os, vSox9, isMorg1, isDhps, isWdr83os, isSox9)
    updateStats(stats["All"],  vMorg1, vDhps, vWdr83os, vSox9, isMorg1, isDhps, isWdr83os, isSox9)
}
```

```
["Podocytes", "PT", "Other", "Unclassified", "All"].each { group ->
    def s = stats[group]

    print "\n--- ${group} ---"
    print "Total cells: ${s.total}"
    print "Sox9+ (nuclear): ${s.sox9_pos} (" + fmt(pct(s.sox9_pos, s.total)) + "%)"
    print "MORG1+: ${s.morg1_pos} (" + fmt(pct(s.morg1_pos, s.total)) + "%)"
    print "DHPS+: ${s.dhps_pos} (" + fmt(pct(s.dhps_pos, s.total)) + "%)"
    print "WDR83os+: ${s.wdr83os_pos} (" + fmt(pct(s.wdr83os_pos, s.total)) + "%)"
    print "Triple M/D/W+: ${s.triple_mdw} (" + fmt(pct(s.triple_mdw, s.total)) + "%)"
    print "Quadruple+: ${s.quadruple} (" + fmt(pct(s.quadruple, s.total)) + "% of all; " + fmt(pct(s.quadruple,
s.sox9_pos)) + "% of Sox9+)"
    print "MORG1+ within Sox9+: ${s.sox9_morg1} (" + fmt(pct(s.sox9_morg1, s.sox9_pos)) + "%)"
    print "DHPS+ within Sox9+: ${s.sox9_dhps} (" + fmt(pct(s.sox9_dhps, s.sox9_pos)) + "%)"
    print "WDR83os+ within Sox9+: ${s.sox9_wdr83os} (" + fmt(pct(s.sox9_wdr83os, s.sox9_pos)) + "%)"
}


["Podocytes", "PT", "Other", "Unclassified", "All"].each { group ->
    def s = stats[group]

    def line = [
        imgName,
        group,
        s.total,

        fmt(mean(s.sum_morg1, s.total)),
        fmt(mean(s.sum_dhps, s.total)),
        fmt(mean(s.sum_wdr83os, s.total)),
        fmt(mean(s.sum_sox9, s.total)),

        s.sox9_pos,
        fmt(pct(s.sox9_pos, s.total)),

        s.morg1_pos,
        fmt(pct(s.morg1_pos, s.total)),

        s.dhps_pos,
        fmt(pct(s.dhps_pos, s.total)),

        s.wdr83os_pos,
        fmt(pct(s.wdr83os_pos, s.total)),

        s.morg1_dhps,
        s.morg1_wdr83os,
        s.dhps_wdr83os,

        s.triple_mdw,
        fmt(pct(s.triple_mdw, s.total)),

        s.quadruple,
        fmt(pct(s.quadruple, s.total)),
        fmt(pct(s.quadruple, s.sox9_pos)),

        s.sox9_morg1,
```

```
        fmt(pct(s.sox9_morg1, s.sox9_pos)),

        s.sox9_dhps,
        fmt(pct(s.sox9_dhps, s.sox9_pos)),

        s.sox9_wdr83os,
        fmt(pct(s.sox9_wdr83os, s.sox9_pos)),

        fmt(mean(s.sum_morg1_in_sox9, s.sox9_pos)),
        fmt(mean(s.sum_dhps_in_sox9, s.sox9_pos)),
        fmt(mean(s.sum_wdr83os_in_sox9, s.sox9_pos)),
        fmt(mean(s.sum_sox9_in_sox9, s.sox9_pos))
    ].join(",")

    outCsv << line + "\n"
}

print "\n→ CSV aktualisiert: ${outCsv.getAbsolutePath()}\n"
```
